# Identification of potential chemosignals in the European water vole *Arvicola terrestris*

**DOI:** 10.1038/s41598-019-54935-z

**Published:** 2019-12-05

**Authors:** Patricia Nagnan-Le Meillour, Amandine Descamps, Chrystelle Le Danvic, Maurane Grandmougin, Jean-Michel Saliou, Christophe Klopp, Marine Milhes, Coralie Bompard, Didier Chesneau, Kevin Poissenot, Matthieu Keller

**Affiliations:** 1 Univ. Lille, CNRS, USC INRA 1409 - UGSF - Unité de Glycobiologie Structurale et Fonctionnelle, F-59000 Lille, France; 2 ALLICE R&D, F-75012 Paris, France; 3 CNRS, INSERM, Univ. Lille, CHU Lille, Institut Pasteur de Lille, Centre d’Infection et d’Immunité de Lille, F-59000 Lille, France; 4 INRA, Sigenae, MIAT, F-31326 Castanet-Tolosan, France; 5 INRA, GeT-PlaGe, Genotoul, F-31326 Castanet-Tolosan, France; 6 INRA, CNRS, IFCE, Univ. Tours, Physiologie de la Reproduction et des Comportements, F-37380 Nouzilly, France

**Keywords:** Chemical ecology, Post-translational modifications, Proteomics, RNA, Small molecules

## Abstract

The water vole *Arvicola terrestris* is endemic to Europe where its outbreak generates severe economic losses for farmers. Our project aimed at characterising putative chemical signals used by this species, to develop new sustainable methods for population control that could also be used for this species protection in Great Britain. The water vole, as well as other rodents, uses specific urination sites as territorial and sex pheromone markers, still unidentified. Lateral scent glands and urine samples were collected from wild males and females caught in the field, at different periods of the year. Their volatile composition was analysed for each individual and not on pooled samples, revealing a specific profile of flank glands in October and a specific profile of urinary volatiles in July. The urinary protein content appeared more contrasted as males secrete higher levels of a lipocalin than females, whenever the trapping period. We named this protein arvicolin. Male and female liver transcript sequencing did not identify any expression of other odorant-binding protein sequence. This work demonstrates that even in absence of genome, identification of chemical signals from wild animals is possible and could be helpful in strategies of species control and protection.

## Introduction

The European water vole *Arvicola terrestris* (=*Arvicola amphibious* L.) is a small rodent belonging to the Cricetidae family, phylogenetically close to the voles of the genus *Microtus*, *Myodes* (bank vole), and to the hamsters *Cricetulus* (Chinese hamster) and *Phodopus* (Siberian hamster). There are two ecological types in *A*. *terrestris*: the amphibious type colonises the banks of the ponds, and river and lake shores. The burrowing type (fossorial) digs subterranean galleries and nests in meadows, pastures and crops. The water vole is protected in England as a threatened species, and paradoxically considered as an agricultural pest in the European continent where its outbreak causes extensive damage to crops and generates severe economic losses to farmers. Indeed, the reproduction period extends from March to October, during which females produce up to 3 litters, each of 4–5 pups^1^. They need to eat 80% of their body weight per day. Their population can reach more than 500 individuals per hectare in the centre of France, in the “Région Auvergne-Rhône-Alpes”. In addition to the damage they cause to crops, they are vectors of tapeworms including *Echinococcus*, responsible for the Human disease echinococcosis, which could be fatal if untreated. In absence of natural predators (wolf, ferret, fox, birds of prey), the main current pest control is based on the use of bromadiolone that has a significant negative environment impact^2^.

There is a need to find new sustainable methods to control the water vole population. Rodents, and particularly murine species, use a very sophisticated chemical-based communication system, exchanging both volatile and protein intraspecific signals^3^. They spread urine and scents from glands secretion^4^ to inform their congeners about their sex and physiological condition^5^, the perception of which induces specific behaviours such as mating^6^, aggression^7,8^, or dominance^9,10^. Urinary proteins, thought to be involved in chemical communication, have been identified in the closely related species *M*. *glareolus*^11,12^. Among them, glareosin is specifically expressed by males^12^ and such a protein in *A*. *terrestris* would be of interest in the objective of mating disruption. Another described sex-dimorphic protein is aphrodisin, a lipocalin secreted in the vaginal discharge of hamster females, a species also related to the water vole, which displays aphrodisiac properties^13,14^. Interestingly, bank vole urinary proteins belong to the Odorant-Binding Protein family^11,12^, and not to the Major Urinary Protein (MUP) family of murine species^15^. Besides urine, the water vole lateral scent gland has been proposed to be a source of chemical signals and its composition has been preliminary determined^16,17^. The sebaceous glands being under androgens control, it has been suggested that the content could be different in and out the reproduction period in males^16^. The aim of this work was to identify molecular cues potentially involved in water vole chemical communication, if possible sex-specific, to use them in field population control. In a first step, we identified the components of the lateral scent gland secretion produced by sebaceous glands after solvent extraction followed by gas chromatography coupled to mass spectrometry (GC/MS). We also identified the volatile components of urine by SPME-GC/MS, and urinary proteins by *de novo* analysis of high-resolution mass spectrometry data, followed by transcriptome sequencing and molecular cloning of encoding sequences from liver RNA. These analyses were performed on individuals, not on pools, either for glands or for urine, to evaluate inter-individual variability in wild-caught animals.

## Results and Discussion

### Lateral scent glands contain volatile compounds and long chain esters of fatty acids

**Figure 1:**
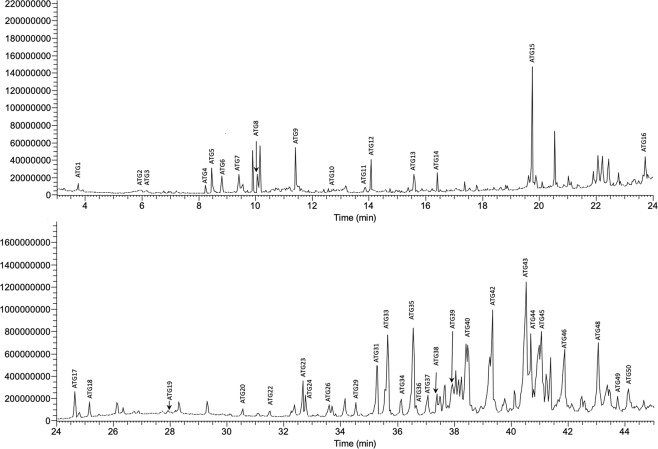
Gas-chromatographic profile of constituents from lateral scent gland (LSG, Male 18 – October) extracted with dichloromethane. Peaks differing from the control were numbered according to retention time (identification in Supplementary Table S1 in electronic file).

**Figure 2:**
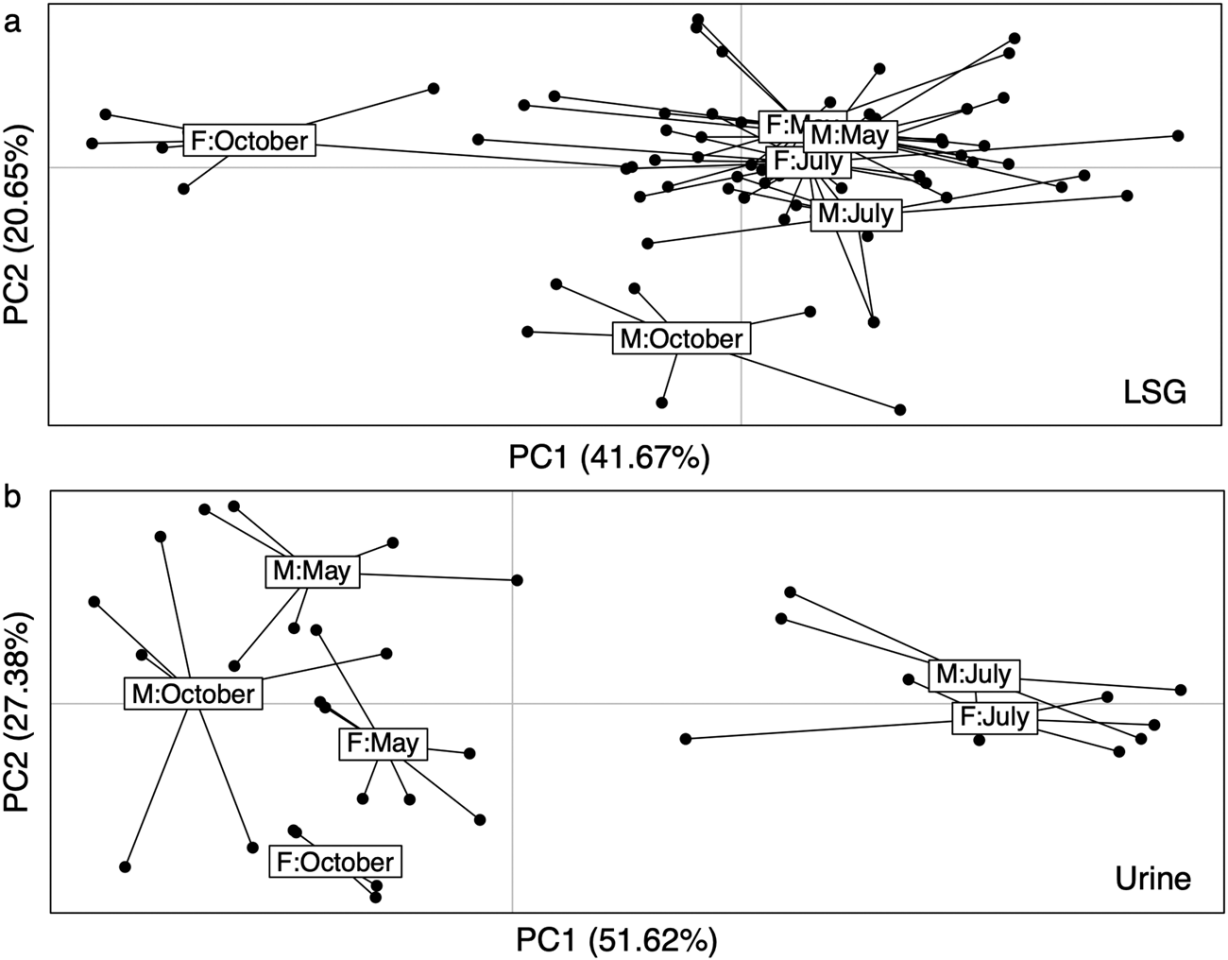
Redundancy Analysis (RDA) score plot from the constraint Principal Component Analysis (PCA) of **(a)** Lateral Scent Gland (LSG) composition and **(b)** urinary volatiles.

Lateral scent glands of *A*. *terrestris* were studied as they were supposed to contribute to the chemical signals exchanged between congeners of this species^16^. A preliminary examination of the chemical content^17^ by GC/MS has suggested the presence of long chain fatty acid esters ranging from C7 to at least C12, cholesterol and long chain alcohols. Chromatograms of solvent extraction of both male and female LSG displayed 50 compounds (Fig. 1), 41 of which were identified (Supplementary Table S1). They were numbered according to their retention time (*A*. *terrestris* glands: ATG-1 to -50). Acids (ATG2, ATG3), ketones (ATG4, ATG6, ATG8), aldehydes (ATG5, ATG9, ATG12, ATG13), two pyrazine derivatives (ATG10, ATG11) and the sesquiterpene caryophyllene (ATG14), constituted the volatile part of the gland content. Besides, a lactone (ATG15), 20 long chain (C15 to C21) esters of acids (C8 to C11) and cholesterol (ATG43) were also identified. When commercially available, authentic chemicals were co-injected to ascertain NIST identification. Long chain esters of acids were identified by manual fragmentation, based on the confident NIST identification of octanoic acid, octadecyl ester (ATG23) that provided the cracking pattern for this family of compounds. Indeed, for the acidic fragment, diagnostic ions were (in *m/z*) 103 for pentanoic, 117 for hexanoic, 145 for octanoic, 159 for nonanoic and 173 for decanoic. For the ester fragment, typical ions (*m/z*) were 224 for hexadecyl, 238 for heptadecyl, 252 for octadecyl, 266 for nonadecyl, 280 for decadecyl, and 294 for undecadecyl. The composition of the LSG was variable between individuals, all compounds were not retrieved in all animals (Fig. 1). A redundancy analysis (RDA) was undertaken to determine whether any variation in LSG composition could be linked to sex and/or month of sampling (Fig. 2a**)**. Our experimental design (the two experimental variables and their interaction taken together) explains only 11.85% of the total variance in the chemical data (p = 0.001) with a significant period of collect effect (p = 0.001). From the PCA and the pairwise comparison, we inferred that the LSG composition did not significantly changed in May and July whatever the sex, partly because of noticeable heterogeneity of chemical profiles. Only the October LSG composition differs from the two other periods (p < 0.0045). The LSG profile slightly stood out in female during October (Fig. 2a, p-value = 0.0033), by the significative absence of four major compounds (ATG36 = hexanoic acid, Xdecyl ester, ATG44, ATG45 = decanoic acid, decadecyl ester, and ATG46 = decanoic acid, undecadecyl ester) compared to May and July. Moreover, October is the only period during which LSG male and female profiles appeared significantly different (p < 0.0033), mainly because of a weak proportion of 6 compounds, unfortunately not all identified (ATG34, ATG44, ATG46, ATG48, ATG49 and ATG50, Kruskal-Wallis, p < 0.05) compared to male profile. The flank organs were described in several vole species, and were proposed to play a role in male dominance^17^, but behavioural data are scarce and contradictory^18^. This work did not evidence a male-specific profile that could support this hypothesis^17^. Only behavioural analyses with LSG secretion could bring information on their role in the water vole chemical communication.

### The water vole specific profile of urinary volatiles

**Figure 3:**
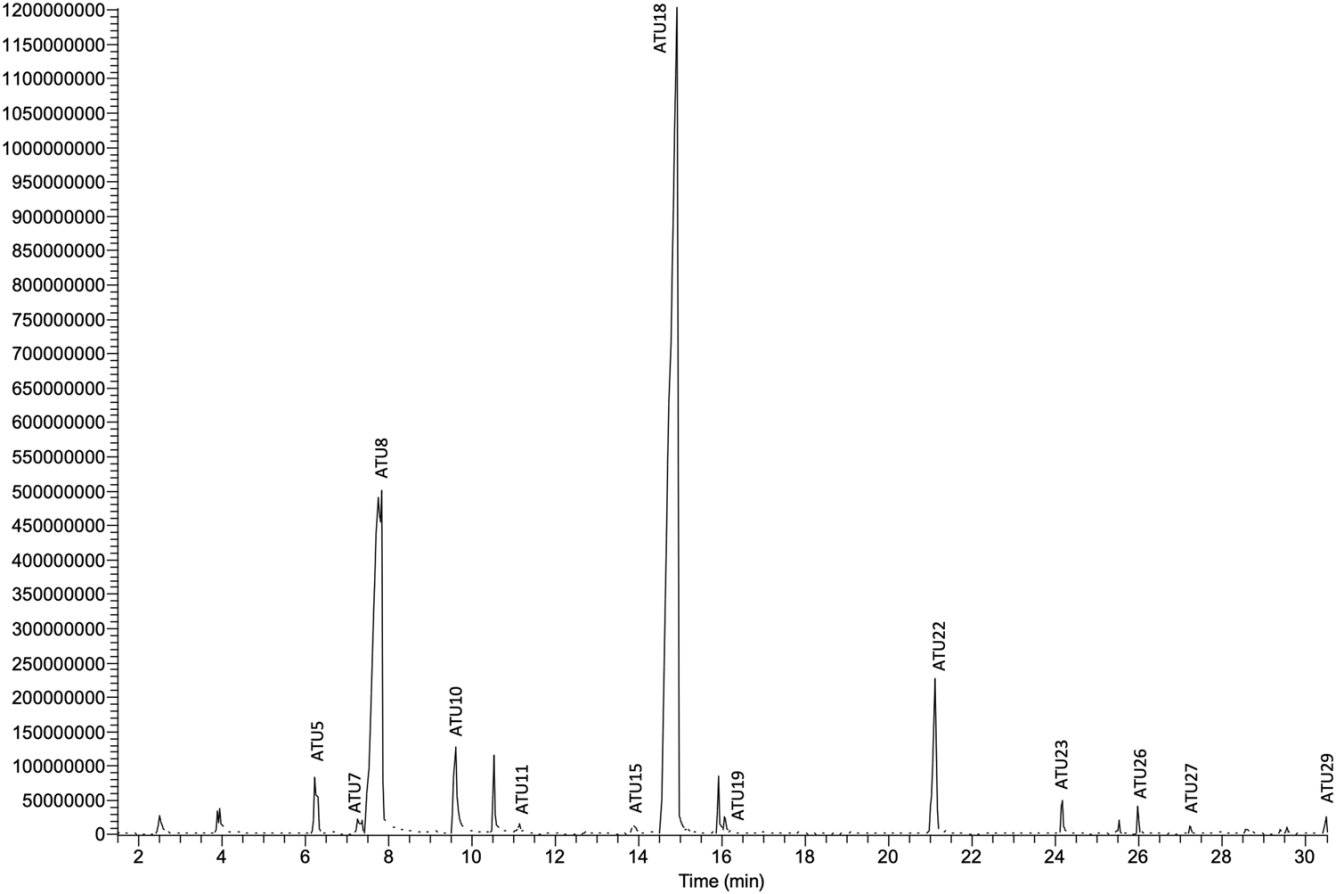
Gas-chromatographic profile of urinary volatiles extracted by SPME (Male 1 - May). Peaks were numbered according to retention time (identification in Supplementary Table S2 in electronic file).

In male and female urine, 29 compounds were detected by SPME-GC/MS and numbered according to the retention time (*A*. *terrestris* urine: ATU1 to ATU29, Fig. 3**)**. Among them, 25 were identified by combined NIST library matches and co-injection of authentic chemicals spectra when commercially available **(**Supplementary Table S2). Identified compounds included ketones, aromatic and pyrazine derivatives. Santene (ATU5), p-Cresol (ATU16) and Coumarin (ATU26) were specific to urine samples collected in July. Most of compounds were retrieved in only one or two animals either in October, or in May, or in July (ATU1-ATU4, ATU6, ATU9, ATU12-ATU14, ATU17, ATU20-21, ATU24, ATU28-29). Conversely, 2,5-dimethyl-pyrazine (ATU8), benzaldehyde (ATU10), 2-methyl-6-(1-propenyl)-, (Z)-pyrazine (ATU18), were present in urine of all animals, whatever their sex and the season. In addition, 1-nitro-3-methylbutane (ATU7), trimethyl pyrazine (ATU11), 3-ethyl, 2,5-dimethyl-pyrazine (ATU15), beta-nitro-styrene (ATU23), alpha-curcumene (ATU26) and ATU27 (unidentified) were retrieved in half of the animals, but were not sex-specific nor season specific. No signal at all was obtained from urine of 6 animals (males and females) caught in October. The mixture of ketones, benzene and pyrazine derivatives, plus a few alcohols and terpenes, composed a water vole specific profile of urinary volatiles, close to those of other Cricetidae species (*Phodopus campbelli and P*. *sungorus*), where pyrazines dominated in number and quantity^19,20^. Indeed, none of the typical mouse species pheromones (for a review see^21^), 2,3-dehydro-exo-brevicomin, 2-sec-butyl-4,5-dihydrothiazole or farnesenes were identified in water vole urine. Urinary volatile compounds of *A*. *terrestris* are closer to Hamster (ketones and pyrazines) urine than to Mouse ones, in accordance with the species phylogeny^22^. In *Phodopus* sp., as in the pine vole *Microtus pinetorum*, males and females also displayed the same profile of ketones and pyrazines, with differences in concentration that authors linked to endocrinological status of animals^19,23^. RDA analysis was performed on peaks area obtained from GC/MS chromatograms of 33 animals (Fig. 2b). Our experimental design explains 23.34% of the total variance in the chemical data (p = 0.002) with independent sex and period effects (p = 0.05 and p = 0.001). Indeed, statistical analysis showed a seasonal difference in both male and female *A*. *terrestris* urinary profiles in July that differed from those observed in May and October (Fig. 2b, p-value = 0.01). The July urinary profile is characterized by a quantitative increase of 3 molecules (ATU5-santene, ATU16-p-cresol, and ATU25-coumarin). Despite a clear heterogeneity in composition, urinary chemical profiles significantly differ between male and female only in May (Fig. 2b, p-value = 0.01). The two major noticeable differences (Kruskal-Wallis, p < 0.05) are 1/the specific presence in male of the two pyrazine derivatives ATU11 (trimethyl-pyrazine) and ATU15 (2,5-dimethyl,3-ethyl-pyrazine) in proportion of 1.47% and 1.35% of the total profile respectively, and 2/twice as much of ATU18 (2-methyl-6-(1-propenyl) (Z)-pyrazine) in male (54.35 ± 14.13%) than in female (21.72 ± 11.24%). While it may be tempting to link these differences to a seasonal breeding, the capture of pregnant females whenever the period rebuts this hypothesis (Supplementary Table S3). Indeed, the lack of sex difference in urinary volatiles was reported in other Cricetidae, such as *Peromyscus californicus*, the California mice^6^ and different *Phodopus* hamster species^19^. Hence, absence of sexual dimorphism in urinary volatile profiles could be unrelated to the outbreak phenomenon, the absence of well-defined breeding season being more likely a characteristic of Cricetidae, as reported for the Prairie vole *M*. *ochrogaster*^24^. This demonstrates the relevance of the individual analysis rather than pools that could smooth the differences. Captures should be done all over the year to give light into this supposed absence of defined reproduction period of the water vole, and/or to confirm seasonal differences with a larger panel of animals.

### Arvicolin is the only lipocalin expressed in water vole urine

**Figure 4:**
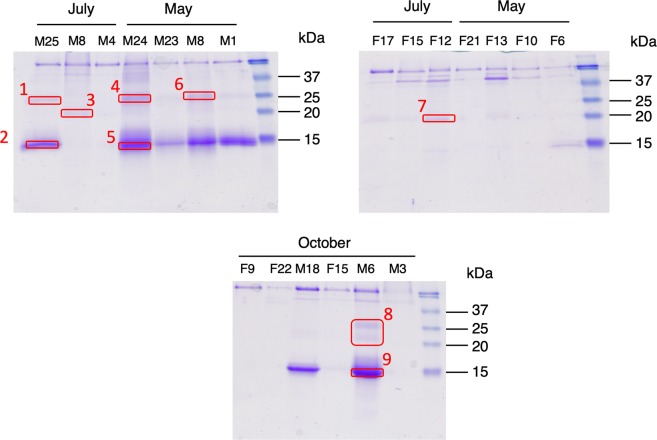
Analysis of urinary protein content of males (M) and females (F) *Arvicola terrestris* caught in the field at different periods of the year (May, July and October). One-dimensional electrophoresis, Coomassie Blue R 250 staining. Molecular weight markers: Precision Plus Protein™ Standards (Bio-Rad). Bands cut out is indicated by red rectangles.

**Figure 5:**
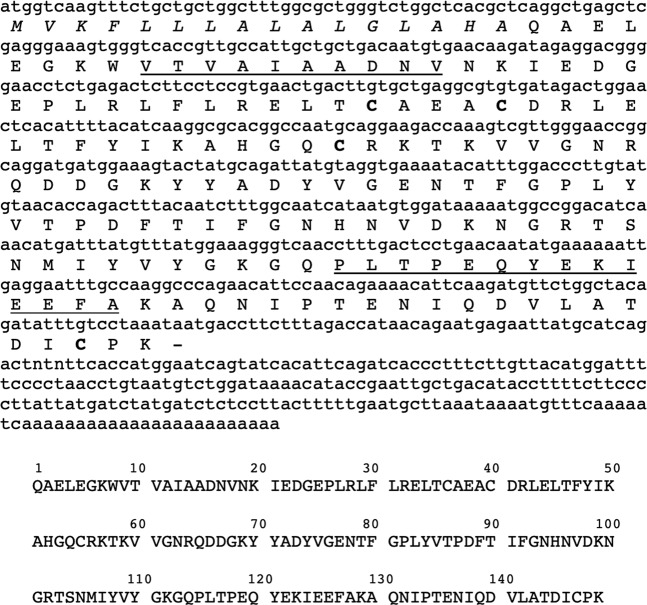
Full-length sequence of *arvicolin* obtained by PCR after 5′- and 3′-RACE. Nucleotide and translated sequence (www.expasy.org/translate/). Signal peptide is in italics. Peptides identified by *de novo* analysis of MS/MS data are underlined. The four cysteines are indicated in bold.

**Figure 6:**
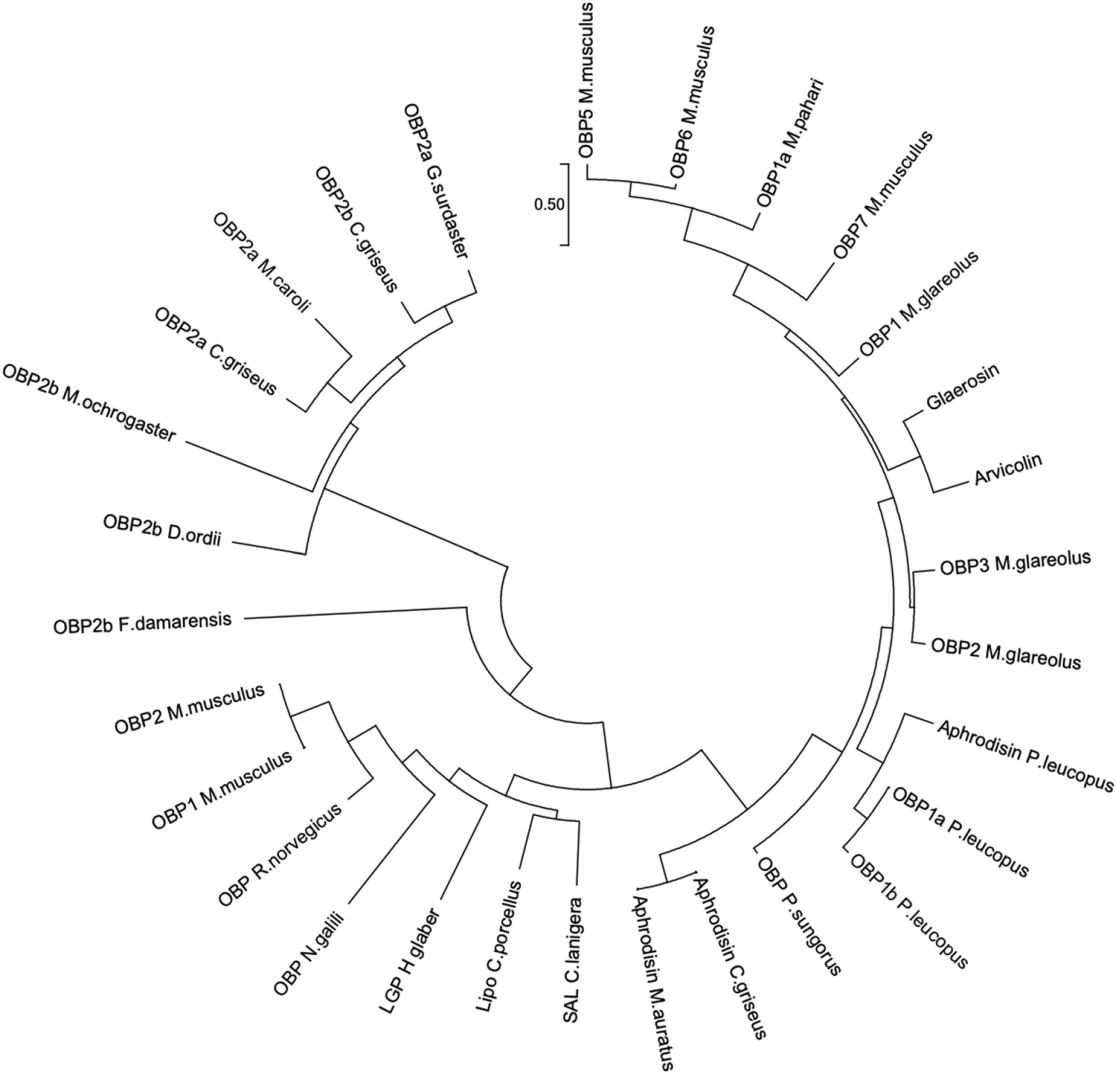
Molecular Phylogenetic analysis by Maximum Likelihood method of OBP-like sequences in the Rodentia order. Sequences used for building the tree can be found online in Supplementary Table S6.

To get a real overview of sex- and season- differences in urinary protein content, we analysed each animal secretion individually and not a pool of animals. Profiles of normalised protein content of urines in 1-DE revealed that males excrete huge quantity of proteins (from 0.2 mg to 1.5 mg/mL urine, value normalised with creatinine), whatever the season, of apparent molecular mass close to 15 kDa (Fig. 4), although males M4 and M8 of July, and M3 of October did not secrete such proteins. Nevertheless, a faint band at a similar position is observed in some female samples (Fig. 4**:** F12 of July, F6 of May, and F15 of October). Bands were cut out gels according to the pattern indicated in Fig. 4 to identify the proteins by nano-LC-MS/MS after trypsin digestion. The water vole genome was not available in any database, but thanks to the closely related species *M*. *glareolus*, peptides of *A*. *terrestris* urinary proteins were blasted by Mascot software and matched to published urinary MglaOBP1, MglaOBP2 and MglaOBP3^11^. A fourth sequence was identified in *M*. *glareolus* urine at the protein level by mass spectrometry, glareosin, as male-specific and restricted to the breeding season^12^. We thus added the glareosin protein sequence in a home-made database for mass spectrometry analyses. Results showed that each band contains peptides, which are homologous to those of MglaOBP-like and glareosin-like proteins (Supplementary Table S4). Few peptides were identified (1 to 3 for each protein) that led to weak percentages of sequence recovery (8–17%), suggesting that *A*. *terrestris* OBP sequences significantly differ from those of *M*. *glareolus*. In order to extend sequence coverage, we used *de novo* analysis to perform an automated interpretation of MS/MS spectra. The quality of the MS/MS spectrum containing the “T-ALAAD” tag was enough to fully annotate the spectrum and to deduce the terminal amino acids according to precursor mass (Supplementary Fig. S1). Similarity with glareosin sequence allowed us to hypothesize that this peptide is part of the N-terminal sequence of a *A*. *terrestris* glareosin-like protein. Besides, the spectrum shown in Supplementary Fig. S2 allowed unambiguous identification of the sequence “PEQYEKLEEF” homologous to MglaOBP2/3^11^ and glareosin^12^ and conserved in *A*. *terrestris*. Then, oligonucleotide primers were designed on the sequence of these two peptides, by using *M*. *glareolus* more frequent codons to increase the possibility to amplify a first part of the glareosin-like. The resulting amplicon was used as template to perform 3′ and 5′ RACE-PCR with specific oligonucleotide primers and GeneRacer™ primers of the GeneRacer™ kit (Supplementary Table S5). The full-length sequence (Fig. 5) encodes a 149 amino acids mature protein (web.expasy.org/translate/) of QAEL N-terminus (Signal P-5.0 server at www.cbs.dtu.dk/services/SignalP/) sharing several features with OBP sequences of closely related Cricetidae species: in addition to the sequences from *M*. *glareolus* (bank vole) OBP and glareosin, and hamster aphrodisin^25^, a search in Ensembl Genome browser (www.ensembl.org) provided OBP sequences from *Microtus ochrogaster* (Northern American Prairie vole), and *Peromyscus maniculatus bairdii* (Northern American deer mouse), which genomes are available (Ensembl.org). These sequences were aligned^26^. The novel protein from *A*. *terrestris* was named “arvicolin” and was deposited in GenBank database under accession number MK984608. Arvicolin shares 69.3% identity (90% similarity) with glareosin and 48.6% (79.1% similar) with aphrodisin. The detailed results obtained by Blastp search are provided in Supplementary Table S6 and were used to construct a phylogenetic tree of OBPs in the Rodentia order (Fig. 6). Arvicolin peptides were identified in bands 2, 5, 9, and also in bands 1, 4, 6, 7 and 8. The fact that the same arvicolin peptides were identified at apparent molecular masses of 15 kDa and 30 kDa is not unusual for OBPs, which form dimers when concentrated, visible even in denaturing conditions^27^.

As mass spectrometry data have indicated other potential sequences related to peptides from MglaOBPs (Supplementary Table S4), and to be confident in the assessment that only arvicolin is expressed in male urine, the liver transcriptome of 3 males and 3 females was sequenced by Illumina. We were able to assemble the full-length sequence of *arvicolin* in all conditions. Aligning 23 genes of Cricetidae species (Supplementary Fig. S3) with MegaBlast on the assemblies indicated that none of these proteins is expressed in males or in females. The number of reads aligned on the *arvicolin* transcript sequence strongly varies between animals. Even if counted in females (Dot plot in Supplementary Fig. S4), the *arvicolin* transcripts are much more numerous in males (from 1,000 for F2 to 50, 000 reads per million reads (FPM) for M4). The female (F2) that expresses the highest number of transcripts was pregnant. The analysis of differentially expressed genes indicated that 325 contigs are differentially expressed between males and females (FDR < 0.05). The sex difference in arvicolin expression is not higher than the difference in expression of any other gene of the set (AT-F7 k43 Locus 18919 Transcript 2.1: log FC = 6.371868, logCPM = 13.73815, LR = 9.77251, P-value = 0.001771405, FDR = 0.1095165). In other words, females secrete less proteins in urine than males. This sex discrepancy has recently been reported for MUP expression in the Norway rat, *Rattus norvegicus*^28^.

### Arvicolin is modified by post-translational modifications

**Figure 7:**
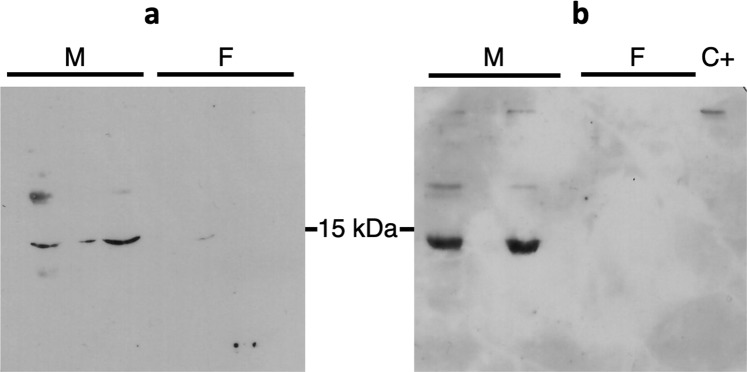
Immunodetection of post translational modifications. (**a**) Western-blot with Q7 antibodies for detection of phospho-threonine-modified proteins, (**b**) Western-blot with CTD110.6 antibody for *O*-GlcNAcylated proteins detection, C+: BSA-*O*-GlcNAc (5 ng).

Arvicolin appears as the single lipocalin secreted in the urine of both sexes, as well as roborowskin of the Roborovski hamster^29^, but much more by males than by females, like glareosin in *M*. *glareolus*^12^. To go further into this hypothesis, urinary protein content of male 6-October was also analysed by 2D-E to separate the proteins potentially co-migrating in the 15 kDa band of 1-DE gels (Supplementary Fig. S5). MS/MS analysis revealed that spots contained the same peptides typical to arvicolin. The spots linear distribution suggested different charges from spot 10 to 18, and a slight molecular mass difference between spots 13-15-17-19 and spots 11-12-14-16. The former difference in charge could be due to phosphorylation of Ser, Tyr, Thr residues, whilst the later could result from the presence of *O*-GlcNAc moieties on Ser and Thr amino acids of the primary sequence. Indeed, these modifications have already been observed on mammalian OBPs^30^ and several sites are predicted for arvicolin by NetPhos 3.1 server and YinOYang 1.2 server (at www.expasy.org). Specific antibodies raised against phosphothreonine (Fig. 7a) and *O*-GlcNAc (Fig. 7b) labelled the band containing arvicolin only in male urinary samples. So, even if expressed, arvicolin does not appear as modified by these two post-translational modifications in female urine, although we cannot totally exclude the fact that their faint quantity in females is under the detection threshold of antibodies. The identification of *O*-GlcNAc sites by mass spectrometry is not possible by using the HCD mode of ionisation, as the energy is too high to conserve the sugar that is very labile. Conversely, two phosphorylated peptides were identified when database searching was performed with phosphorylation as variable modification. Comparison between the fragmentation profiles of the phosphorylated peptide and the corresponding naked peptide, the shift in retention time and the *m/z* of the precursors, clearly confirmed the identification (Supplementary Fig. S6). Two phosphate groups were detected on peptide 103–123 (TSNMIYVYGKGQPLTPEQYEK), but the fragmentation did not allow to precise the exact position of the phosphorylation between Thr103, Ser104 and Tyr108 (both predicted by NetPhos), and Tyr110 (spectra in Supplementary Fig. S6a,b). A third phosphorylation was identified in C-terminal peptide 130–149 on Thr13 (predicted by NetPhos server, spectra in Supplementary Fig. S6c,d). To summarize, the phosphorylation of arvicolin is supported by the presence of isoforms distributed in a gradient of charge in 2D-E gels, immunoreactivity to anti-phosphothreonine antibodies, and identification of potential phosphosites by mass spectrometry. Phosphorylated peptides from rat urinary proteins have been identified at a consensus site for FAM20C^28^, an enzyme that phosphorylates numerous secreted proteins^31^. There is no such site in arvicolin, no more than in porcine OBP sequence where phosphorylation sites were unambiguously localised however^30^, nor in ovine and caprine OBPs^32^, nor in Giant Panda OBP4^33^, also phosphorylated. Thus, if the report of phosphorylation of OBP and related proteins is increasing^27,28,30,32,33^, the molecular pathway and enzymes involved are far from to be understood. Conversely, the *O*-GlcNAcylation of OBPs is less reported, although very interesting as a regulating key of OBP binding specificity to VOCs or pheromone components^30,32^. The presence of *O*-GlcNAc on arvicolin is ascertained by (1) the high specificity of CTD110.6 antibodies and (2) the fact that arvicolin is the only protein localised in the labelled band in males. The sex-difference in phosphorylation and *O*-GlcNAcylation has been proposed to be governed by hormones^27,30,32^.

### Molecular modelling of arvicolin 3-dimensional structure

**Figure 8:**
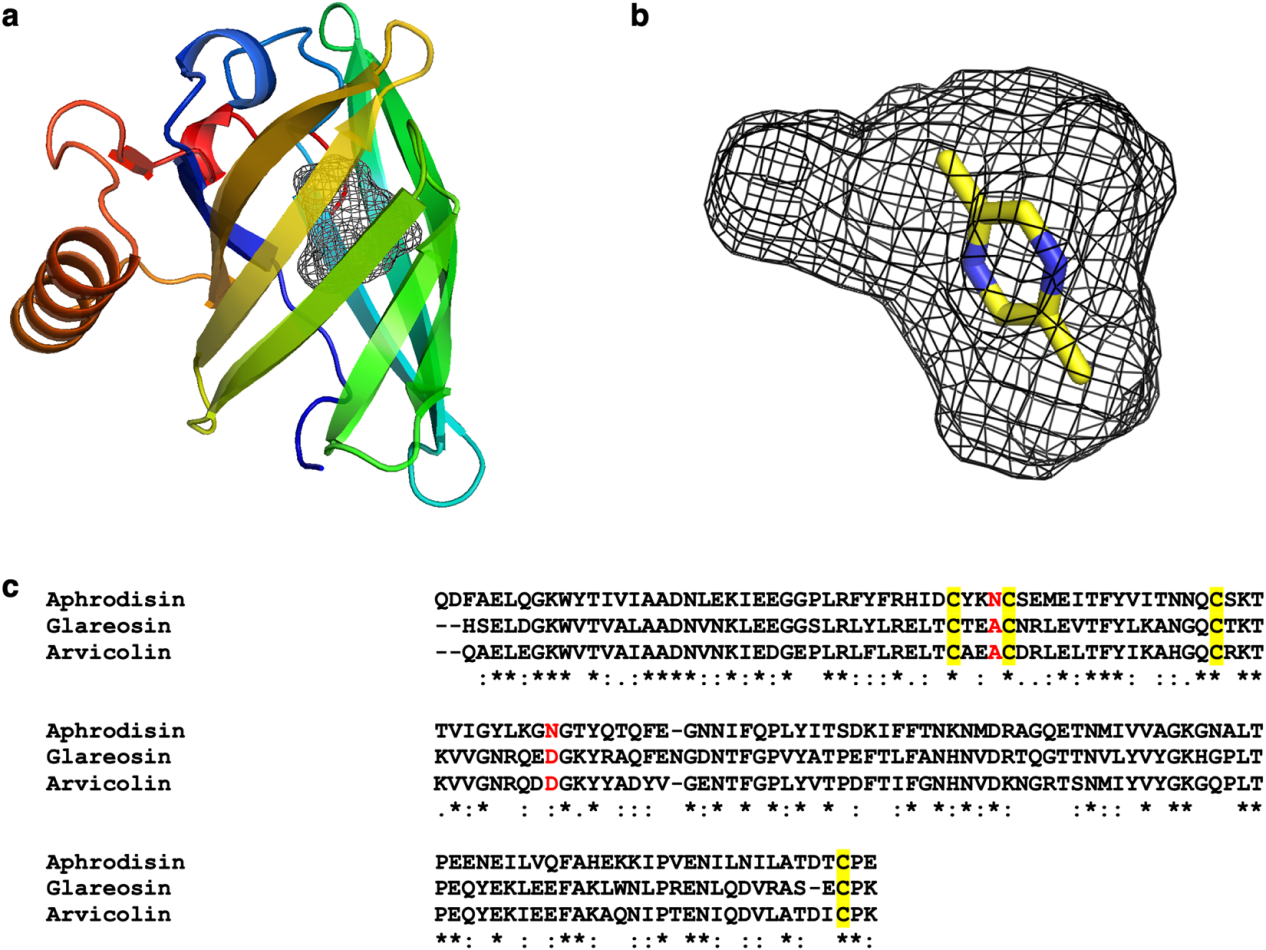
3-D structure of arvicolin predicted by homology modelling. (**a**) The model is coloured blue to red from N-terminus to C-terminus and is represented by secondary structure elements. The cavity inside the β-barrel is represented by black mesh. The figure was generated using PyMOL software (The PyMOL Molecular Graphics System, Version 1.8.0.0 Schrödinger, LLC, https://pymol.org/2/), (**b**) Structure of 2,5-dimethyl pyrazine docked in arvicolin cavity, (**c**) CLUSTAL O (1.2.4) multiple sequence alignment of aphrodisin (sp|P09465.3|APHR_CRICR), glareosin^12^, and arvicolin (GenBank# QEY02203.1). Conserved cysteines are underlined in yellow, glycosylation sites of aphrodisin are red bold (N, Asn) and corresponding amino acids (A, Ala, and D, Asp) in glareosin and arvicolin are in red.

Three-dimensional structure prediction has shown that arvicolin displays a classical lipocalin fold (Fig. 8a) also observed for aphrodisin of female hamster^34^ and the model of glareosin^12^. As observed in the structure of aphrodisin, the walls of the barrel cavity are formed by aromatic and aliphatic residues and three polar non-charged residues. However, despite the very high sequence and structure homology between these proteins, the central β-barrel cavity of arvicolin estimated by GHECOM^35^ is 233 Å^3^ which is 90 Å^3^ less than glareosin and aphrodisin cavities, suggesting that arvicolin may transport smaller molecules. Meanwhile, docking simulations between pig salivary lipocalin (SAL), another OBP, and its specific ligands, have demonstrated an extend on the cavity volume upon ligand binding^36^. This phenomenon could happen for arvicolin, but the size of its cavity is large enough to accommodate the small pyrazine derivatives forming the signature of the water vole urine, such as pyrazine-2,5-dimethyl (Fig. 8b). A first disulphide bridge Cys55-Cys147 is conserved in all lipocalins^37^. The second disulfide bridge Cys36-Cys40, specific to the pheromone transporter sub-class of lipocalins (PBP) of rodents is conserved, but the two N-glycosylation sites occupied in aphrodisin are not present in arvicolin, nor in glareosin. In these sequences, Asn41 and Asn69 of aphrodisin are replaced by Ala39 and Asp 67 respectively (Fig. 8c). N-glycosylation sites are located at the opposite face of the cavity entrance and have been proposed to be involved in the interaction with receptors of aphrodisin^34^. Interestingly, aphrodisin, a female protein secreted by uterus cells into the vaginal discharge and transferred to the male during copulation is N-glycosylated^13,14^. Arvicolin, secreted by liver cells into urine of both sexes is not N-glycosylated, but could be modified by phosphorylation and *O*-GlcNAcylation. These different post-translational modifications are probably linked to different kinds of internal ligands and receptors, depending on their role in chemical communication of Cricetidae. Indeed, PTM could generate isoforms with different binding affinities to ligands as reported for porcine OBP^30^. Thus, albeit small, the binding pocket could accommodate the different ligands identified in water vole urine, in particular pyrazine derivatives, the binding of which could be governed by isoforms specificity.

### Volatile, semi-volatile and protein molecules identified could act as chemosignals in the water vole chemical communication

Most of the compounds identified in LSG or urine were described as players in chemical communication of animals, mainly rodents^38^. Among them, pyrazine-2,5-dimethyl is an adrenal-dependent molecule that delays puberty in female mice^39^, and also in the Cricetidae *P*. *californicus*^6^. The ubiquitous presence of this molecule in male and female urine whatever the season does not preclude its behavioural impact in combination with other urinary molecules, of volatile or protein nature. Although the composition of urinary volatile was stable across seasons and sexes, some variations occurred that could reflect slight differences in urine composition linked to animals’ hormones, and thus, to different effects on the behaviour. In rodent species, it is largely documented that odorants are bound by urinary proteins^40^ that slowly and timely release their content to the air once urine is laid on the ground^10^. Unfortunately, quantities of urine collected for each animal were not compatible with assays to dissociate odorants from urinary proteins with heat or pH changes^41^ for monitoring eventual modifications of the volatile urinary profile. Our study on wild animals of different physiological status cannot bring relevant information of the fine significance of these chemosignals. Meanwhile, the identification of potential chemosignals of the water vole *A*. *terrestris* paves the way to behavioural analyses that will define the pheromonal activity of these volatile and non-volatile compounds.

## Methods

### Animals and sampling

Wild males and females were caught in France, in a land parcel of experimental INRA domain of Marcenat (Latitude 46.2338, longitude 3.3979) and Laqueuille (Latitude 45.65, longitude 2.7333) in a zone of water vole *A*. *terrestris* outbreak. Urine was sampled by natural miction or by soft massage of the pubis directly above glass vials. For each animal, body, and testis or uterus weight were registered in a laboratory close to the field. Control of parasite (echinococcosis) was assessed by visual examination of the liver, and infected animals were not included in this study. Lateral scent glands (LSG) were also excised, put in a glass vial by pair for each animal and stored at −80 °C in 2 mL dichloromethane. Samples of soil were collected as control. Three campaigns were run in October 2016 (8 males and 12 females), May (10 males and 16 females) and July 2017 (8 males and 17 females), to link the data to the reproduction period. A fourth campaign run in April 2018 was dedicated to liver dissection for RNA extraction (3 males and 3 females). Data are summarized in Supplementary Table S3. The animals trapping is authorised in departments of Cantal and Puy-de-Dôme by prefectoral orders 2015–1381 and 2015–155 that can be found as Supplementary Information 2 electronic file. Collection of samples on animals was performed in accordance with directive 2010/63/EU.

### Analysis of LSG and urine content by GC/MS

Analyses were not conducted on LSG or urine pools, but on each animal sample. Lateral scent glands and urine compounds were identified by GC/MS in a FOCUS DSQII (Thermo Fisher Scientific) equipped with a GC capillary column SLB^®^-5MS (30 m × 0.25 mm ID × 0.25 μM, Supelco) under carrier gas helium flow of 1 mL/min. LSG samples (dichloromethane) were filtered on 0.22 μM filters and the filtrate was evaporated under gentle nitrogen stream until 250 μL. One μL was injected in the GC/MS in splitless mode (15 s) and the temperature program was 40 °C to 200 °C in 5 °C/min, then 200 °C to 290 °C in 20 °C/min. Each sample was analysed in triplicate. Urine (100 μL in 2 mL glass vial) was thawed immediately before volatile extraction by SPME (Solid-Phase MicroExtraction) onto a 50/30 μM DVB/CAR/PDMS fiber (Supelco), 45 min at 50 °C under bar magnet agitation at 200 rpm. Before the first analysis and between analyses, the fibre was conditioned by heating in the GC injection port at 260 °C during 3 min, following manufacturer’s instructions. The fibre was desorpted into the GC/MS and compounds were separated according to the following program: Splitless 3 min, 40 °C to 255 °C in 10 °C/min, 255 °C to 315 °C in 5 °C/min, 315 °C to 340 °C in 2 °C/min. Carrier gas was helium at a 1 mL/min flow. Samples were analysed in triplicate.

Compounds were identified by comparison of spectra with those of the implemented National Institute Search Technology NIST98 library using Excalibur software (Thermo Fisher Scientific), and by manual analysis of fragmented ions in spectra. Then, identification was confirmed by comparison to authentic standard spectrum obtained by co-injection in the same GC/MS conditions, when commercially available. Each sample was injected three times and relative quantification of each compound was determined by peak area value. Raw data were subjected to Principal Component Analysis (PCA) by using the package FactoMineR of the R software (R 3.5.1 GUI 1.70) to discriminate biologically meaningful clusters. A comparison using a non-parametric Kruskal-Wallis test was also performed. Statistical analyses data were performed using the R software (R 3.5.1 GUI 1.70). Data were subjected to Redundancy analysis (RDA) to discriminate biologically meaningful clusters using the script provided by Hervé *et al*. (Example 2)^42^ and 3 R packages (“Hotelling”, “Vegan”, “RVAdeMemoire”). Briefly, the analysis consisted in two steps: 1/fitting a multivariate linear regression between chemical data and the experimental variables (sex and period) followed by 2/a principal component analysis (PCA). To access the effect of our experimental variables on observed chemical variations, a permutation F-test based on the canonical R^2^ was used. Individual effect of experimental variables and their interaction was achieved using a similar permutation F-test. Comparison using non-parametric tests (Mann and Whitney and Kruskal-Wallis) were also performed.

### Analysis of urinary proteins

#### Extraction and separation of proteins by 1-dimensional (1-DE) and 2-dimensional electrophoresis (2-DE)

Proteins were extracted from 100 μL urine by phase partition with 300 μL cold CH_3_Cl/MeOH (2/1) on ice. After brief mixing in Vortex, samples were centrifuged (15,000 g for 15 min at 4 °C). The methanol phase was collected and evaporated in a Speed-Vac concentrator (Eppendorf). Dried proteins were stored at −20 °C. To correct for differences in urine dilution, creatinine concentration was determined by using the Creatinine Assay Kit (Sigma-Aldrich) on 100 μL of urine for each animal, in triplicate. Normalised quantities of proteins were separated on 1-DE gels in conditions already described^43^, and 2-DE was performed as already published^30^.

#### Immunodetection of post-translational modifications

After 2-DE, gels were either stained with colloidal Coomassie blue R solution (12% trichloroacetic acid, 5% ethanolic solution of 0.035% Serva blue R-250) or transferred onto PVDF membranes (Mini-PVDF transfer pack, Bio-Rad) in the Trans-Blot Turbo Transfer system (Bio-Rad) according to manufacturer’s instructions. For immunodetection of *O*-GlcNAc modified proteins, western-blot was performed with CTD110.6 antibody (1:5,000 dilution, Sigma-Aldrich) as already published^28^. Phosphorylated proteins were detected using anti-phospho-Threonine antibodies (Q7 at 1:500, Qiagen) according to manufacturer’s protocol (Phosphoprotein Purification kit, Qiagen).

#### Identification of urinary proteins by high-resolution mass spectrometry and “de novo” analysis

Spots were excised from 1-DE or 2-DE gels, destained and in-gel proteins were reduced, carbamidomethylated and digested by trypsin. Peptides were extracted and automatically fractionated onto an Ultimate 3000 RSLC nano system with a commercial C18 reverse phase column (75 µm × 150 mm, 2-µm particle). Elution was performed at a flow rate of 300 nL/min and a gradient separation from 5% to 25% ACN and 0.1% FA during 12 min. The eluted peptides were analysed by a Q-Exactive Orbitrap scanning in full MS over *m/z* 300–1200 range with a resolution of 35000 at *m/z* 200. Three most intense peaks with charge state between 2 and 4 were fragmented in the HCD collision cell with normalised collision energy of 35%, and tandem mass spectra were acquired with a resolution of 17500 at *m/z* 200. Dynamic exclusion was set to 7 s.

Raw data collected during nano-LC-MS/MS analyses were processed and converted into *.mgf peak list format with Proteome Discoverer 1.4. MS/MS data were interpreted using search engine Mascot (version 2.4.0) installed on a local server. Searches were performed with a tolerance on mass measurement of 0.2 Da for precursor and 0.2 Da for fragment-ion, against a composite target decoy database built with *M*. *glareolus* UniProt database (taxon 447135, May 2018, 1311 entries) fused with the sequence of *M*. *glareolus* glareosin protein^12^, recombinant trypsin and a list of classical contaminants (119 entries). For each sample, peptides were filtered out according to the cut-off set for proteins hits with one or more peptides taller than nine residues, ion score >10, identity score >4, and 1% false positive rate. All tandem mass spectra were also sequenced by *de novo* pipeline of MSDA platform (10.1002/pmic.201300415). Same both search parameters and database than automated Mascot searches were applied for *de novo* sequencing and homology searches. All interesting spectra were manually annotated according *de novo* tag identification, precursor mass and *M*. *glareolus* protein sequences.

#### RNA extraction and molecular cloning of glareosin-like sequence

The liver of 3 males (AT-M1, AT-M3, AT-M4) and 3 females (AT-F2, AT-F7, AT-F8) was resected and immediately put into RNAlater (QIAGEN). Total RNA was extracted from 30 mg liver of each animal with the RNeasy Universal Mini kit containing Qiazol Lysis Reagent (QIAGEN). Purity of RNA was checked in a BioPhotometer D30 (Eppendorf). Reverse transcription was performed using the Superscript IV First-Strand Synthesis System (Invitrogen) with 3′ specific primer designed from mass spectrometry “*de novo*” sequencing with *M*. *glareolus* OBP codons as template (all primers mentioned in this paragraph can be found in Supplementary Table S5). PCR (AccuPrime™ *Pfx* SuperMix, Invitrogen) with primers designed from *de novo* analysis allowed identification of a portion of glareosin-like sequence. Then, 5′ and 3′RACE-PCR (Rapid Amplification of cDNA Ends-Polymerase Chain Reaction) were run to get the full-length mRNA sequence of *A*. *terrestris* glareosin-like. The GeneRacer kit was used according to manufacturer’s protocol (Invitrogen), with a slight modification at RNA clean up steps, which were performed with the RNeasy MinElute CleanUp kit (QIAGEN). At all steps, amplicons were cloned using Zero-blunt^®^TOPO^®^ PCR Cloning Kit for sequencing (Invitrogen) and were sequenced in both senses (eurofins Genomics). A final amplification from 5′-end to 3′-end of the glareosin-like sequence was performed from RNA liver of the six animals, and the amplicons were cloned and sequenced in the same conditions as described above.

#### RNA sequencing and data analysis

RNA-seq libraries (same 3 males and 3 females as above) have been prepared according to Illumina’s protocols using the Illumina TruSeq Stranded mRNA sample prep kit to analyse mRNA. Briefly, mRNAs were selected using poly-T beads, then fragmented to generate double stranded cDNA. Adaptors were ligated to be sequenced after 11 cycles of PCR to amplify libraries. Library quality was assessed using Fragment Analyzer and libraries were quantified by qPCR using the Kapa Library Quantification kit. RNA-seq experiments have been performed on an Illumina HiSeq 3000 device using a paired-end read length of 2 × 150 pb with the Illumina HiSeq 3000 sequencing kits.

The six conditions were individually assembled with runDrap for DRAP (version 1.91)^44^ using Oases with the standard multiple kmer approach. Contig files were merged with DRAP runMeta (version 1.91) using standard parameters. Because in conditions where *arvicolin* was highly expressed the assembly produced only partial transcripts, the corresponding samples read files were down-sampled and re-assembled. All sample files produced complete *arvicolin* transcripts. Only one *arvicolin* reference contig was present in the merged contig file. Reads have been realigned on the contigs with bwa mem (version 0.7.12-r1039) using standard parameters. Alignment files have been compressed, sorted, indexed and quantified with Samtools view, sort, index and Idxstats (version 1.3.1) with standard parameters. The count file has been produced with Unix cut and paste tools. The analysis of differentially expressed genes was performed with edgeR version 3.26.1.

#### Molecular Phylogenetic analysis by Maximum Likelihood method

The evolutionary history was inferred by using the Maximum Likelihood method based on the JTT matrix-based model^45^. The tree with the highest log likelihood (−4331.33) is shown. Initial tree(s) for the heuristic search were obtained automatically by applying Neighbor-Join and BioNJ algorithms to a matrix of pairwise distances estimated using a JTT model, and then selecting the topology with superior log likelihood value. The tree is drawn to scale, with branch lengths measured in the number of substitutions per site. The analysis involved 29 amino acid sequences (Supplementary Table S6). All positions containing gaps and missing data were eliminated. There was a total of 106 positions in the final dataset. Evolutionary analyses were conducted in MEGA7^46^.

#### Homology modelling

The model of the structure of arvicolin was calculated using I-TASSER^47^. Only one model was generated with a C-score of 1.01. Among the top 10 identified structural analogues in the protein data bank (PDB) used to generate the model, arvicolin shared 47.9% identity with aphrodisin of female hamster (PDB entry code 1E5P, 10.2210/pdb1E5P/pdb)^34^ and 98% with the model superimposed with 1E5P, at a root mean square deviation of 1.26 Å.

### Ethical statement

The animals trapping is authorised in departments of Cantal and Puy-de-Dôme by prefectoral orders 2015-1381 and 2015-155 that can be found as Supplementary Information 2 electronic file. Collection of samples on animals were performed in accordance with directive 2010/63/EU.

## Supplementary information

Supplementary Information1

Supplementary Information2

## Data Availability

The GC/MS spectra of lateral scent glands and urine content generated during this study will be provided to any qualified researcher upon request. The mass spectrometry proteomics data have been deposited to the ProteomeXchange Consortium via the PRIDE^48^ partner repository with the dataset identifier PXD013803 and 10.6019/PXD013803. The full-length nucleotide sequence of arvicolin was deposited in GenBank database under accession number MK984608. Raw data coming from the sequencing by Illumina of the liver transcriptome of the six animals were deposited in the sequence Read Archive of NCBI under Bioproject #PRJNA545200.
