# Supplementary material for: Identification of potential chemosignals in the European water vole Arvicola terrestris
Source: Sci Rep. 2019 Dec 5;9:18378. doi: 10.1038/s41598-019-54935-z (PMC6895148; doi:10.1038/s41598-019-54935-z)

Identification of potential chemical signals in the European water vole *Arvicola terrestris*

Patricia Nagnan-Le Meillour, Amandine Descamps, Chrystelle Le Danvic, Maurane Grandmougin, Jean-Michel Saliou, Christophe Klopp, Marine Milhes, Coralie Bompard, Didier Chesneau, Kevin Poissenot, Matthieu Keller

Supplementary information 1: supplementary figures and tables

(presented in order of appearance in the text)

Supplementary Table S1. Identified and partially identified (*italics*) compounds in *A. terrestris* male and female (N=68) lateral scent glands by GC/MS

Supplementary Table S2. Identified and partially identified (*italics*) compounds in *A. terrestris* male and female urine (N= 33) by SPME-GC/MS

Supplementary Table S3. Animals caught in the field used for identification of urinary volatiles and proteins, and RNA sequencing

Supplementary Table S4. Peptides identified in bands from 1D-E gels of males and females *A. terrestris* (pattern in Figure 4), which are homologous to peptides of *M. glareolus* OBPs

Supplementary Figure S1. MS/MS spectrum of the WVTVALAADNVNK peptide

Supplementary Figure S2. MS/MS spectrum of the GQPLTPEQYEKLEEFAK peptide

Supplementary Table S5. Primers used for amplification of the sequence encoding glareosin-like (arvicolin) from *A. terrestris* male liver

Supplementary Table S6. Sequences from Blastp search with arvicolin protein sequence as query and used for the building of the phylogenetic tree (Fig. 6)

Supplementary Figure S3. Sequences used in Megablast search

Supplementary Figure S4. Number of arvicolin reads counted in the liver transcriptome of *A. terrestris* males and females

Supplementary Figure S5. Two-dimensional electrophoresis of male 6 (October) extract of urinary proteins

Supplementary Figure S6. MSMS spectra of identified phosphopeptides from arvicolin.

Supplementary Table S1. Identified and partially identified (*italics*) compounds in *A. terrestris* male and female (N=68) lateral scent glands by GC/MS (NIST = NIST library; Std = commercial compound) - * refers to compounds not present in the chromatogram of **Fig. 1** (Male 18 – October), but identified in other animals.

Name	Rt (min)	Compound	Identification	Name	Rt (min)	Compound	Identification	Name	Rt (min)	Compound	Identification
ATG1	03.75	3-ethyl, hexane	NIST + Std	ATG18	25.16	1-heptadecanol	NIST	ATG35	*36.47*	*Xdecyl ester, hexanoic acid*	Fragmentation
ATG2	05.96	3-methyl, butanoic acid	NIST	*ATG19*	*27.72*	*1-octadecanol*	*NIST*	*ATG36*	36.55	nonadecyl ester, octanoic acid	
ATG3	06.18	2-methyl, butanoic acid	NIST + Std	ATG20	30.52	pentadecyl ester, octanoic acid	Fragmentation	ATG37	37.06	nonadecyl ester, octanoic acid	Fragmentation
ATG4	08.23	6-methyl, 2-heptanone	NIST	*ATG21**	31.45	*Xdecyl ester (?), nonanoic acid*	Fragmentation	ATG38	37.38	nonadecyl ester, nonanoic acid	Fragmentation
ATG5	08.46	benzaldehyde	NIST + Std	*ATG22*	*31.50*	*3-methyl-, hexadecyl ester(?), butanoic acid*	*Fragmentation*	ATG39	37.92	decadecyl ester, octanoic acid,	Fragmentation
ATG6	*08.81*	*4-methyl-5,6-dihydropyran-2-one*	*See ATG8 spectrum*	ATG23	32.67	hexadecyl ester, octanoic acid	Fragmentation	ATG40	38.40	octadecyl ester, decanoic acid,	Fragmentation
ATG7	09.42	2-methyl-3-methylene, nonane	NIST	ATG24	32.75	unidentified compound 3		ATG41*	38.75	decadecyl ester, nonanoic acid	Fragmentation
ATG8	10.06	5,6-dihydro-2H-Pyran-2-one	NIST	ATG25*	33.01	hexadecyl ester, pentanoic acid	Fragmentation	ATG42	39.24	nonadecyl ester, decanoic acid	Fragmentation
ATG9	11.40	nonanal	NIST + Std	ATG26	33.60	heptadecyl ester, octanoic acid,	Fragmentation	ATG43	40.52	(-)-Cholesterol	NIST
ATG10	12.72	2-isoamylpyrazine	NIST	ATG27*	34.14	unidentified compound 4		ATG44	40.68	unidentified compound 5	
ATG11	13.90	2-isoamyl-6-methylpyrazine	NIST	*ATG28**	34.16	*1-heneicosyl formate with 2 double bonds*	NIST	ATG45	41.07	decadecyl ester, decanoic acid	Fragmentation
ATG12	14.07	2-Decenal, (Z)-	NIST + Std	ATG29	34.53	heptadecyl ester, pentanoic acid	NIST	ATG46	41.87	undecadecyl ester, decanoic acid	Fragmentation
ATG13	15.58	undecenal	NIST + Std	ATG30*	35.06	octadecyl ester, octanoic acid	Fragmentation	ATG47*	43.02	unidentified compound 6	
ATG14	16.41	caryophyllene	NIST + Std	*ATG31*	35.26	*Octadecenol (?)*	Fragmentation	ATG48	43.05	unidentified compound 7	

**ATG15**	19.74	codecalactone	NIST library	**ATG32***	35.51	hexadecyl ester, decanoic acid	Fragmentation	**ATG49**	43.72	unidentified compound 8	
**ATG16**	23.72	unidentified compound 1		**ATG33**	35.61	octadecyl ester, octanoic acid (isomer of ATG30?)	Fragmentation	**ATG50**	44.05	unidentified compound 9	
**ATG17**	24.65	unidentified compound 2		**ATG34**	36.13	heptadecenyl ester, nonanoic acid	Fragmentation				

Supplementary Table S2. Identified and partially identified (*italics*) compounds in *A. terrestris* male and female urine (N= 33) by SPME-GC/MS (NIST = NIST library; Std = commercial compound) - * refers to compounds not present in the chromatogram of **Fig. 3** (Male 1 – May), but identified in other animals

Name	Rt (min)	Compound	Identification	Name	Rt (min)	Compound	Identification
**ATU1***	01.95	1-penten-3-one	NIST + Std	**ATU16***	14.29	4-methylphenol (p-Cresol)	NIST
**ATU2***	02.04	3-pentanone	NIST	***ATU17****	*14.54*	*6-Propenyl bicyclo[3.1.0] hexan-2-one*	*NIST*
**ATU3***	02.10	heptane	NIST +Std	**ATU18**	14.67	2-methyl-6-(1-propenyl)-, (Z)- pyrazine	NIST
**ATU4***	02.53	3-penten-2-one	NIST + Std	**ATU19**	15.30	nitrile benzyl	NIST + Std
**ATU5**	06.24	2,3-dimethyl, bicyclo[2.2.1]hept-2-ene (santene)	NIST	***ATU20****	*16.05*	*2,6,6-Trimethyl-2-cyclohexene-1,4-dione*	*NIST*
**ATU6***	06.69	styrene	NIST + Std	**ATU21***	17.88	octanoic acid	NIST + Std
**ATU7**	07.27	1-nitro-3-methylbutane	NIST	***ATU22***	*21.12*	*di(2-phenylethyl) ester, oxalic acid*	*NIST*
**ATU8**	07.81	2,5-dimethyl-pyrazine	NIST + Std	**ATU23**	24.15	2-nitroethenyl, benzene (beta-nitro-styrene)	NIST
**ATU9***	08.23	dimethyl sulfone	NIST + Std	**ATU24***	24.55	Unidentified compound 2	
**ATU10**	09.61	benzaldehyde	NIST + Std	**ATU25***	24.96	2H-1-benzopyran-2-one (Coumarin)	
**ATU11**	11.15	trimethyl pyrazine	NIST + Std	**ATU26**	25.96	1-(1,5-dimethyl-4-hexenyl)-4-methyl, benzene (Alpha-curcumene)	NIST
**ATU12***	11.54	Unidentified compound 1		**ATU27**	27.95	Unidentified compound 3	
**ATU13***	12.30	2-hydroxy benzaldehyde	NIST	**ATU28***	27.56	Unidentified compound 4	
**ATU14***	12.53	acetaldehyde benzene	NIST + Std	***ATU29***	*30.48*	*1,1'-oxybis, octane*	*NIST*
**ATU15**	13.87	3-ethyl-2,5-dimethyl, pyrazine	NIST				

Supplementary Table S3. Animals caught in the field used for identification of urinary volatiles and proteins, and RNA sequencing

- no urine collected
- only glands were collected, not enough urine for SPME
- proteins were analysed (see Fig. 1)
- only RNA from liver was analysed

2016 October   Marcenat

Animal #	Sex	Weight (g)	Urine	LSG	Testis weight (mg)	Uterus weight (mg)	Proteins	Volatiles	RNA	Vaginal swab
1	M	79.7	X		356.6			no signal		
3	M	55	X	X	106.1			X		
5	F	61.1	X					no signal		
6	M	94.7	X	X	203.2			X		
7	F	63.7	X	X		102.3				
8	F	62.8		X		7.4				
9	F	74.3	X	X		49.9		no signal		
10	M	71		X	248.9			no signal		
11	F	108.7		X		172				
12	M	44.5	X	X	50.3					
13	F	75.4		X		36.2				
15	F	96.4	X	X		72.4		no signal		
16	M	88.3		X	225.9					
17	F	84.1		X		164.1				
18	M	65.2	X	X	332.1			X		
19	F	78.2		X		121.5				
20	M	70.5	X		145.4			no signal		

21	F	83.5		X		129.6		
22	F	65.9	X	X		91.7	X	
23	F	97.6		X		264.8		Start of pregnancy
2017 May		Laqueuille						
1	M	141	X	X	494.9		X	
2	F	116		X		223.5		diestrus
3	F	122		X				pregnant
4	F	94	X	X		220.5	X	estrus
5	M	56.1		X	136.1			
6	F	122	X	X		258.9	X	
7	F	38.3	X	X		26.7		
8	M	121	X	X	556.1		X	
9	F	122.4		X		212.1		metestrus
10	F	127	X	X		185.8	X	metestrus
11	F	58		X		96.3		estrus
12	F	43.7		X		31.9		
13	F	89.9	X	X		244	X	estrus
14	M	87	X	X	523			
15	M	64	X	X	173.4		X	
17	F	40		X		38.5		
18	M		X	X	400.2			
19	F	114		X				pregnant
20	F	53.5		X		89.7		estrus
21	F	114	X	X			X	pregnant
22	F	122	X	X		147.8	X	metestrus
23	M	131.6	X	X	498.7		X	
24	M	119	X	X	612.1		X	

25	M	69.9	X	X	181.1			X	
26	M	126		X	589.1				
28	F	106	X	X		303.6		X	estrus
2017 July		Laqueuille							
2	F	44.8	X	X		59		X	
3	F	77.9		X		61.5			diestrus
4	M	41.8	X	X	62.3			no signal	
5	F	90.6	X	X		186.1		X	diestrus
6	M	25.9	X	X	41.1				
7	F	24.6		X		12			
8	M	27.8	X	X	57			X	
9	F	60.2		X		98.4			metestrus
10	F	24.3	X	X		11.5			
11	M	65.2		X	344				
12	F	89.6	X	X		164		X	metestrus
13	F	96		X					pregnant
14	M	25.2	X	X	38			X	
15	F	97.4	X	X		173.2		X	diestrus
16	F	108.5		X					pregnant
17	F	92.9	X	X				X	pregnant
18	M	138.9	X	X	442.7			X	
19	F	142		X					pregnant
20	F	85		X		92			
21	F	90.3		X		295.4			diestrus
22	M	61.4		X	230				
23	F	55.7		X		49.2			diestrus
24	F	76.6		X					pregnant

25	M	114.2	X	X	520		X	
26	F	104.8		X		211		diestrus
2018 April		Saint-Julien-Puy-Lavèze						
1	M	73			323.1			
2	F	130						End of pregnancy
3	M	132			493.4			
4	M	122			392.1			
7	F	108				304.6		Diestrus
8	F	84				144.4		Estrus

Supplementary Table S4. Peptides identified in bands from 1D-E gels of males and females *A. terrestris* (pattern in Figure 4), which are homologous to peptides of *M. glareolus* OBPs. Band # is indicated in Fig.4. Peptides are replaced (in red) on the *M. glareolus* sequences below.

Protein name	Protein accession number	Calculated molecular mass	Band #	Peptide (s)	% of sequence coverage
Glareosin	Not available. Sequence in Loxley *et al.* (2017) supp. data	16933	1 to 9	WVTVALAADNVNK-TQGTTNVLYVYGK	9-17
MglaOBP1	D3VW62_MYOGA	16646	1, 2, 4, 5, 9	DLLATDTCPE-WVTTAIAADNVDK	8-14
MglaOBP2	D3VW63_MYOGA	16840	1 to 9	GQPLTPEQYEK-GQPLTPEQYEKLEEFAK-LIFVVGKGQPLTPEQYEK	10-15
MglaOBP3	D3VW64_MYOGA	16752	1 to 9	EQNIPTENIR-GQPLTPEQYEK-GQPLTPEQYEKLEEFAK	10-16

```
Glaerosin       HSELDGKWVTVALAADNVNKLEEGGSLRLYLRELTCTEACNRLEVTFYLKANGQCTKTKV    60
D3VW62_MYOGA    QAELEGKWVTTAIAADNVDKIERGGPLRLYIRKITCTEACSKMEVTFYVNENNQCSQTKI    60
D3VW63_MYOGA    QAELEGKWVTTAIAADNIDTIEEEGPMRIYVRELTCSEACSKMGVTFYVNANGQCSETKV    60
D3VW64_MYOGA    YAELEGTWYTTAIAADNVDTIEEEGPLRLYVRELTCSKGCNKLGVTFYVNANGQCSKTTV    60

Glaerosin       VGNRQEDGKYRAQFENGDNTFGPVYATPEFTLFANHNVDRTQGTTNVLYVYGKHGPLTPE    120
D3VW62_MYOGA    TGYRQEDGNYRAQFE-GDNVFKPVAATEDIIVFASENVDRAGRTTNLVLVAGKGQPLTPE    119
D3VW63_MYOGA    IGYRQEDGKYRTQFE-GDNRFEPVHATPENIVFTNKNVDRTGRTTKLIFVVGKGQPLTPE    119
D3VW64_MYOGA    TGYMQEDGKYRTQFE-GDDRFKPVHATPDNIVFISQNVDRAGRTTNLIFVVGKGQPLTPE    119

Glaerosin       QYEKLEEFAKLWNLPRENLQDVRAS-ECPK    149
D3VW62_MYOGA    QHEKLEAYAKEHNIPPENIRDLLATDTCPE    149
D3VW63_MYOGA    QYEKLEEFAKEQGIPTENIREVLPTDTCPE    149
D3VW64_MYOGA    QYEKLEEFAKEQNIPTENIRNVLATDTCPE     149
```

Supplementary Figure S1. MS/MS spectrum of the WVTVALAADNVNK peptide. *De novo* analysis allows identifying the tag sequence "T-ALAAD" in ion 700.88 m/z 2^+-charged. Manual annotation confirms the presence of the tag and completes sequencing with clear identification of the y2 fragment, corresponding to the dipeptide KN and allowing to deduce the dipeptide VN – the fragments y9 and y10 identifying Valine as the missing amino acid into the tag T-ALAAD – and the fragments y11 and y12 allowing to identify the WV dipeptide according to the measured mass of the peptide (1399.76 Da).

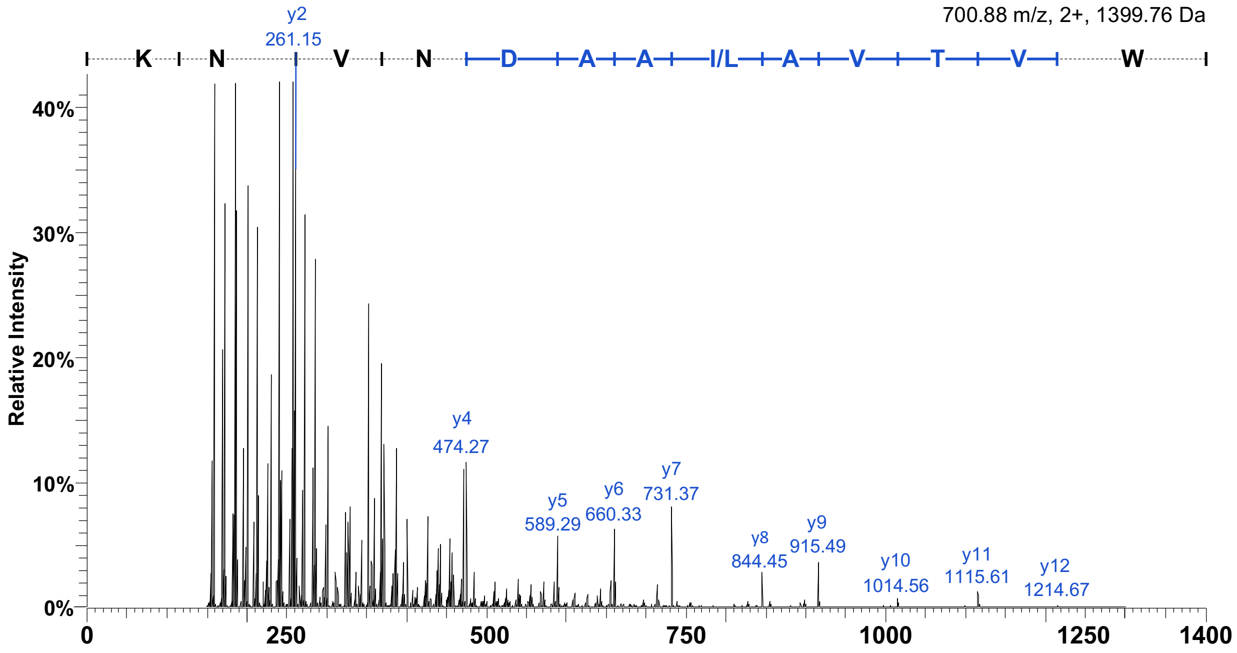

Supplementary Figure S2. MS/MS spectrum of the GQPLTPEQYEKLEEFAK peptide. Database searching allowed identifying the ion of m/z 669.67 (3+) as matching with GQPLTPEQYEKLEEFAK peptide mass with a precursor mass error of 1.3 ppm and an ion score of 70.9. Upper red and blue sequences indicate the amino acids validated by detection of respectively N-terminal and C-terminal peptide fragments (b and y ions series).

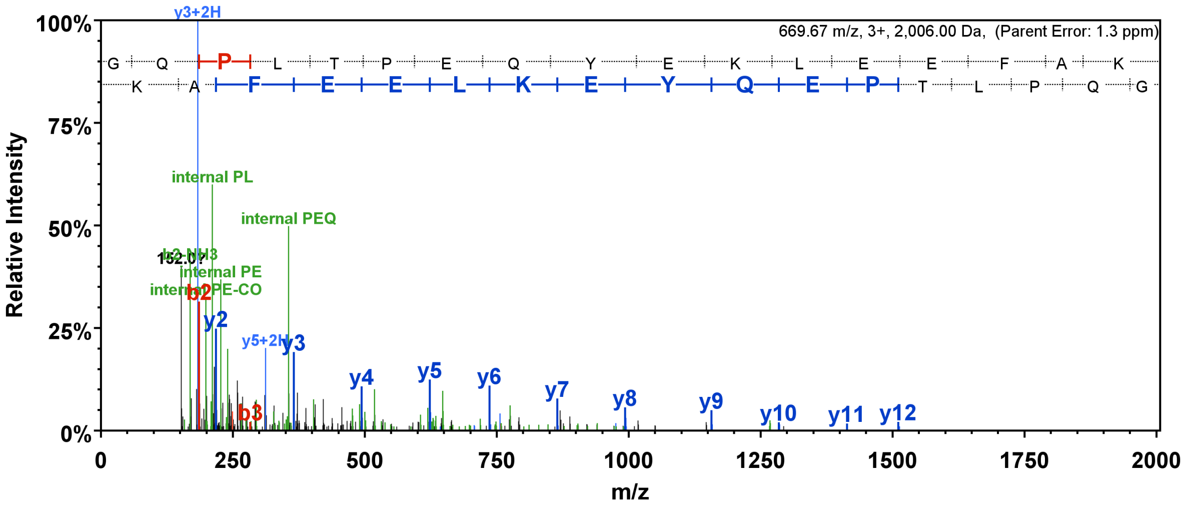

Supplementary Table S5. Primers used for amplification of the sequence encoding glareosin-like (arvicolin) from *A. terrestris* male liver

	5’ primer (forward)	3’ primer (reverse)
PCR with *de novo* MS information	Peptide WVTVALAADNV at N-ter position<br>5’-TGGGTTACCGTTGCCCTTGCTGCTGACAATGTG-3’	Peptide PLTPEQYEKLEEFA at C-ter position<br>5’-CCTTTGACTCCTGAACAATATGAAAAACTTGAGGAATTTGCC-3’
5’RACE PCR	GeneRacer™ 5’ primer<br>5’-CGACTGGAGCACGAGGACACTGA-3’	Gene Specific 5’ primer<br>5’-CCCGTCCTCTATCTTGTTCACATTGTCAGCAGC-3’
5’RACE nested PCR	GeneRacer™ 5’ Nested Primer<br>5’-GGACACTGACATGGACTGAAGGAGTA-3’	Gene Specific 5’ nested primer<br>5’-TCTATCACACGCCTCAGCACAAGTCAGTTCA-3’
3’RACE PCR	Gene Specific 3’ primer<br>5’-GTTACCGTTGCCCTTGCTGCTGACAATGTGAAC-3’	GeneRacer™ 3’ primer<br>5’-GCTGTCAACGATACGCTACGTAACG-3’
Full-length amplification from animal liver	5ARVICOLIN<br>5’-ATGGTCAAGTTTCTGCTGCTGGCTT-3’	3ARVICOLIN<br>5’-TTATTTAGGACAAATATCTGTAGCCAGAACATC-3’

Supplementary Table S6. Sequences used for the building of the phylogenetic tree (Fig. 6)

Accession	Description	Name in the tree
lcl|Query_9764	arvicolin	arvicolin
QEY02203	glareosin	glareosin
P09465	aphrodisin[Cricetulus griseus]	Aphrodisin_C. griseus
Q9Z1I7	Aphrodisin Mesocricetus auratus	Aphrodisin_M.auratus
NP_620258.1	odorant-binding protein precursor [Rattus norvegicus]	OBP_R.norvegicus
AIA65156.1	odorant binding protein 1 [Mus musculus musculus]	OBP1_M.musculus_musculus
AIA65157.1	odorant binding protein 2 [Mus musculus musculus]	OBP2_M.musculus_musculus
AIA65158.1	odorant binding protein 5 [Mus musculus musculus]	OBP5_M.musculus_musculus
AIA65159.1	odorant binding protein 6 [Mus musculus musculus]	OBP6_M.musculus_musculus
AIA65155.1	odorant binding protein 7 [Mus musculus domesticus]	OBP7_M.musculus
XP_005346787.2	odorant-binding protein 2b [Microtus ochrogaster]	OBP2b_M.ochrogaster
ADC97771.1	odorant binding protein 2 [Myodes glareolus]	OBP2_M.glareolus
ADC97770.1	odorant binding protein 1 [Myodes glareolus]	OBP1_M.glareolus
ADC97772.1	odorant binding protein 3 [Myodes glareolus]	OBP3_M.glareolus
XP_028719045.1	aphrodisin-like [Peromyscus leucopus]	Aphrodisin _P. leucopus
XP_028717128.1	odorant-binding protein 1a-like [Peromyscus leucopus]	OBP1a_P. leucopus
XP_028717126.1	odorant-binding protein 1b-like [Peromyscus leucopus]	OBP1b_P. leucopus
AGT28425.1	odorant-binding protein [Phodopus sungorus]	OBP_P. sungorus
XP_027278553.1	odorant-binding protein 2a isoform X2 [Cricetulus griseus]	OBP2a_C.griseus
EGW10044.1	Odorant-binding protein 2b [Cricetulus griseus]	OBP2b_C.griseus
XP_005406585.1	PREDICTED: male-specific submandibular salivary gland protein-like [Chinchilla lanigera]	SAL_C.lanigera
XP_028639070.1	odorant-binding protein 2a-like [Grammomys surdaster]	OBP2a_G.surdaster
XP_021044251.1	odorant-binding protein 1a-like [Mus pahari]	OBP1a_M.pahari
XP_021012716.1	odorant-binding protein 2a [Mus caroli]	OBP2a_M.caroli
XP_012876753.1	odorant-binding protein 2b [Dipodomys ordii]	OBP2b_D.ordii
KFO21503.1	Odorant-binding protein 2b [Fukomys damarensis]	OBP2b_F.damarensis
XP_010604442.1	PREDICTED: female-specific lacrimal gland protein [Fukomys damarensis]	LGP_F.damarensis
XP_004867396.1	female-specific lacrimal gland protein-like [Heterocephalus glaber]	LGP_H.glaber
VEV85354.1	allergen lipocalin Cav p 1 isoform 2 precusrsor [Cavia porcellus]	Lipo_C.porcellus

Supplementary Figure S3. Sequences used in Megablast search

1. ***Microtus ochrogaster***

>ENSMOCT00000026459.1 Moch1
EGKWDTIAVAADNVAKIEEGGPLRLYARELTCSEECNKLEVTF
YVNANGQCSETKITGYRQEDGTYRTQYEGDGRFKPVHGISDNIVFICQNVDRAGQTTNLI
YVIGKGQPLTPEQYEKLEEFAKEQNMPTENIRNVLATDTCPQ

>ENSMOCT00000020380.1 Moch2
YTELEGAWFTTAIAADNVDTIEEEGPMRLYVRELTCSEACNEMD
VTFYVNANGQCSETTVTGYRQEDGKYRTQFEGDNRFEPVYATSENIVFTNKNVDRTGRTT
NQIFVVGKGQPLTPEQYEKLEEFAKQQNIPKENIRQVL

>ENSMOCT00000021279.1 Moch3
ELEGKWVTTAIAADNVDKIEEGGPMRFYMRELTCCEECSQME
ITFYVNVNDQCSQTKITAYRQEDGNYRTQFEGDHVFKPVFATEDIIVLAGDNVDRASRRT
KLIFVLEPLTPEQHEKLEAYVEERSIPPENIRDVLATDTFPK

>ENSMOCT00000011433.1 Moch4
YAELGGKWLTTAIAADNVDKIEKEGPLRLYVREITCSEACSQMG
VTFYVNANGQCSATKVIAYRQEDGKYRTQFEGDNRFEPVHATPENIVFTSKNVDRAGQTT
NLIFVVGKGKPLNPEQYEKLEEFAKEQNIPKENIREVLAT

>XP_005358711.1 aphrodisin-like [Microtus ochrogaster]
HAEIEGKWVTTAIAADNADIIEEGQPLRLYVRELACTEACNRLKFTFYTKVNGQ
CTKTKVIANRQADDQYRARFEGDNIIVPVYVTPEIIIFVVQNVNRTGRKTNLIYVLGKGQSLTSVQYEKLEEFAK
VQNIPRKNIQDILATDICPK

2. **Hamsters**

>CAA46342.1 aphrodisin precursor protein [Cricetus cricetus]
QDFAELQGKWYTIVIAADNLEKIEEGGPLRFYFRHIDCYKNCSEMEITFYVITN
NQCSKTTVIGYLKGNGTYQTQFEGNNIFQPLYITSDKIFFTNKNMDRAGQETNMIVVAGKGNALTPEENEILVQF
AHEKKIPVENILNILATDTCPE

>CAA12414.1 aphrodisin [Mesocricetus auratus]
QDFAELQGKWYTIVIAADNLEKIEEGGPLRFYFRHIDCYKNCSEMEITFYVITN
NQCSKTTVIGYLKGNGTYQTQFEGNNIFQPLYITSDKIFFTNKNMDRAGQETNMIVVAGKGNALTPEENEILVQF
AHEKKIPVENILNILATDTCPE

3. ***Myodes glareolus***

>Glaerosin
HSELDGKWVT VALAADNVNK LEEGGSLRLY LRELTCTEAC NRLEVTFYLK ANGQCTKTKV
VGNRQEDGKY RAQFENGDNT FGPVYATPEF TLFANHNVDR TQGTTNVLYV YGKHGPLTPE
QYEKLEEFAK LWNLPRENLQ DVRASECPK

>ADC97770.1 odorant binding protein 1 [Myodes glareolus]
QAELEGKWVTTAIAADNVDKIERGGPLRLYIRKITCTEACSKMEVTFYVNENNQ
CSQTKITGYRQEDGNYRAQFEGDNVFKPVAATEDIIVFASENVDRAGRTTNLVLVAGKGQPLTPEQHEKL
EAYAKEHNIPPENIRDLLATDTCPE

>ADC97771.1 odorant binding protein 2 [Myodes glareolus]
QAELEGKWVTTAIAADNIDTIEEEGPMRIYVRELTCSEACSKMGVTFYVNANGQ
CSETKVIGYRQEDGKYRTQFEGDNRFEPVHATPENIVFTNKNVDRTGRTTKLIFVVGKGQPLTPEQYEKL
EEFAKEQGIPTENIREVLPTDTCPE

>ADC97772.1 odorant binding protein 3 [Myodes glareolus]

YAELEGTWYTTAIAADNVDTIEEEGPLRLYVRELTCSKGCNKLGVTFYVNANGQ
CSKTTVTGYMQEDGKYRTQFEGDDRFKPVHATPDNIVFISQNVDRAGRTTNLIFVVGKGQPLTPEQYEKL
EEFAKEQNIPTENIRNVLATDTCPE

4. *Peromyscus maniculatus bairdii*

>ENSPEMP00000028399.1 pep
primary_assembly:HU_Pman_2.1:X:97146138:97150695:1
gene:ENSPEMG00000023943.2 transcript:ENSPEMT00000032820.2
gene_biotype:protein_coding transcript_biotype:protein_coding
description:aphrodisin-like [Source:NCBI gene;Acc:102925781]
TMVKFLLLALAFGLAHAYAELQGKWKTTAAAADNVEKIEKGGNLRLYVREVTCNHDCTEM
YITFYVNLNGQCSKTTVTGYKQEDGTYKTQFEGDNVFQPVYATSKNIVFTGKNTDRAGKE
TKLIFVLGKGGSLNEEENEKLEEFTEKEGIPKENIRDVLATDTCPK

>ENSPEMP00000016619.2 pep
primary_assembly:HU_Pman_2.1:X:97981442:97985253:1
gene:ENSPEMG00000015794.2 transcript:ENSPEMT00000020931.2
gene_biotype:protein_coding transcript_biotype:protein_coding
MVKFLLLALALGVSCAHHNNPEITPSEVDGNWRTLYIGADNVEKVLKGGPLRAYFQHMEC
SDECQTLTITFKVKVEGECQTHTVVGRKEKDGLYMTDYSGKNYFRVIEKADGIIIFHNVN
VDNSGKETNVILVAGKEESLSKAQKQELKKLANKLNIPNENIQHLEPADTCHQ

>ENSPEMP00000032118.1 pep
primary_assembly:HU_Pman_2.1:X:98056579:98060489:-1
gene:ENSPEMG00000027218.1 transcript:ENSPEMT00000034930.1
gene_biotype:protein_coding transcript_biotype:protein_coding
TMVKFLLLALAFGLAHAHAEVDGKWETVAIAADNVDKIEVERPLRLYLRELTCNEECSEM
GVTFYVKESVFFKYFHFTDEGDNKYELVYLTEEHAVFTSKNVDRAGTETNLIFVLGKGGA
LNEEEHEKLVKFAEDKNIPTENIRNVLATGNINENFLAISYMCVCVLMYINKYMYAYI

>ENSPEMP00000029229.1 pep
primary_assembly:HU_Pman_2.1:X:98057327:98060486:-1
gene:ENSPEMG00000027218.1 transcript:ENSPEMT00000037177.1
gene_biotype:protein_coding transcript_biotype:protein_coding
MVKFLLLALAFGLAHAHVDGKWETVAIAADNVDKIEVERPLRLYLRELTCNEECSEMGVT
FYVNRLFMFLIFKYFHFTDEGDNKYELVYLTEEHAVFTSKNVDRAGTETNLIFVLGKNGS
GLSENVAKVSLCKRGFRQIQPQIQYHGISEFLSTK

>ENSPEMP00000011691.1 pep
primary_assembly:HU_Pman_2.1:X:98684305:98693100:1
gene:ENSPEMG00000012253.2 transcript:ENSPEMT00000015881.2
gene_biotype:protein_coding transcript_biotype:protein_coding
gene_symbol:Pbsn description:probasin [Source:MGI Symbol;Acc:MGI:1860484]
TMRVFILLLMLDLLGVSSVMMNKNLRKKIQGNWRTVYLASSTVEKISVDSPLRTYFRRIE
CGKRCRQIHFYFYIKKGARCQQYEVIGKKRQEFYQAQYEGKITVMIKMVNDKILLFHYFN
KDVRGKVTRVAGVLARARKLTKDEMTQYMDLVEEIGIEDENVQRVLDTDTCP

>ENSPEMP00000011694.1 pep
primary_assembly:HU_Pman_2.1:X:98684305:98693100:1
gene:ENSPEMG00000012253.2 transcript:ENSPEMT00000015884.2
gene_biotype:protein_coding transcript_biotype:protein_coding
gene_symbol:Pbsn description:probasin [Source:MGI Symbol;Acc:MGI:1860484]
TMRVFILLLMLDLLGVSSVMMNKNLRKKIQGNWRTVYLASSTVEKISVDSPLRTYFRRIE
CGKRCRQIHFYFYIKKGARCQQYEVIGKKRQEFYQAQSRARKLTKDEMTQYMDLVEEIGI
EDENVQRVLDTDTCP

>ENSPEMP00000010512.1 pep
primary_assembly:HU_Pman_2.1:4:14484302:14486186:-1
gene:ENSPEMG00000011475.2 transcript:ENSPEMT00000014687.2
gene_biotype:protein_coding transcript_biotype:protein_coding

gene_symbol:Lcn9 description:lipocalin 9 [Source:MGI
Symbol;Acc:MGI:1924954]
MVVLLVLGLVPSLAAAQFNWEIISQKNYNMAKISGIWYSIFMASDNMTRIEENGDLRVFM
RNINPLKNGSLKFDFFFMVHGECVAVTMVCEKTENYGEFTVAYEGENRVLLTETDYRMYI
TFYMQNIKNGTKTHVLALYGRLPVLSSSYLERFVNICKKYRLNAQNIIDLTNKDVCF

>ENSPEMP00000019617.1 pep
primary_assembly:HU_Pman_2.1:4:14522300:14525589:-1
gene:ENSPEMG00000017847.2 transcript:ENSPEMT00000023958.2
gene_biotype:protein_coding transcript_biotype:protein_coding
gene_symbol:Lcn11 description:lipocalin 11 [Source:MGI
Symbol;Acc:MGI:2684955]
IPGRHSRGMKLLLLLSVGLGLAWTLQDFSPEQVIGSWETLKLASNDRSVVEEEGAYECFM
TGIALLDNGNLNVSYFHRKDGKYVKEFYVAEKTDTPGRYTFEYHGKNYLTFVAVTEEFAI
IDLENQRDGEPLIVVELHGRTQEEGKLGMAHYRRQTARRGILPSNIVDVFLKRPCATQE

>ENSPEMP00000011685.1 pep
primary_assembly:HU_Pman_2.1:4:14571108:14574294:-1
gene:ENSPEMG00000012251.2 transcript:ENSPEMT00000015875.2
gene_biotype:protein_coding transcript_biotype:protein_coding
gene_symbol:Obp2b description:odorant-binding protein 2b-like [Source:NCBI
gene;Acc:102925381]
MKNLLIFLLLGLVAVLKAQEVPSDDQEELSGTWHIKALVCDKNHTEREGPKKVFPMTVTA
LEGGDLEVEITFWKKGQCHKKKIVMHKTDEPGKYTAFKGKKVIYIQELSVKDHYIFYCEG
QHHGKSRRMGKLVGRNPEENPEALEEFKKFAQGKGLRQENILVPEQSDQCTPESD

>ENSPEMP00000027785.1 pep
primary_assembly:HU_Pman_2.1:2:81215874:81218970:-1
gene:ENSPEMG00000023493.2 transcript:ENSPEMT00000032202.2
gene_biotype:protein_coding transcript_biotype:protein_coding
MKLLLMLLLLALELTLVCVHAEGETSARGKNFNPKKIEGKWYSVGLASDKREMIEEGGSM
RVFVEDIDVFENSSLAFKFHTKINGVCTGLYLVCDETEKDGEYFVEYDGRNKFTVLDTDY
DGYVIFHLRNVNKGETFQLMELYGRKQDLSSKIKEKFAELCKKHGIVEENILDLTEADRC
LQARDG

>ENSPEMP00000035169.1 pep
primary_assembly:HU_Pman_2.1:2:81264179:81265670:1
gene:ENSPEMG00000031411.1 transcript:ENSPEMT00000041420.1
gene_biotype:protein_coding transcript_biotype:protein_coding
LVCDETEKDGEYFVEYDGRNKFTVLDTDYDGYVIFHLRNVNKGETFQLMELYGRKQDLSS
KIKEKFAELCKKHGIVEENILDLTEADRCLQARDG

>ENSPEMP00000012546.2 pep
primary_assembly:HU_Pman_2.1:2:82263569:82269126:-1
gene:ENSPEMG00000012837.2 transcript:ENSPEMT00000016754.2
gene_biotype:protein_coding transcript_biotype:protein_coding
gene_symbol:Ambp description:alpha 1 microglobulin/bikunin precursor
[Source:MGI Symbol;Acc:MGI:88002]
MQNLKTQRPQSHAAPPEKAHPAETLPDIKGQHNGNTSRQTGIYGKWFNLAIGSTCPWLRK
IKDKISMSTLVLQEGETEAEISVSSTRWRRGVCEEISWTYEKTDIDGKFLSYNPKWNATL
ETYVVHTNYDEYAIFITKKFTRDHGPTITAKLYGREPQLRDSLLLEFREVALSVGIPENS
IIFMVDRGKCQQSVDGPHPWLWVIRRRPSTKCSKAQNTLFLRV

Supplementary Figure S4. Number of arvicolin reads counted in the liver transcriptome of *A. terrestris* males and females. Raw data coming from the sequencing by Illumina of the liver transcriptome of the six animals were deposited in the sequence Read Archive of NCBI under Bioproject #PRJNA545200.

Sex	Animal	reads by million of sequences
Female	AT-F2	745,7882
Female	AT-F8	15,82129
Female	AT-F7	79,36758
Male	AT-M1	101,3807
Male	AT-M3	36710,23
Male	AT-M4	44792,08

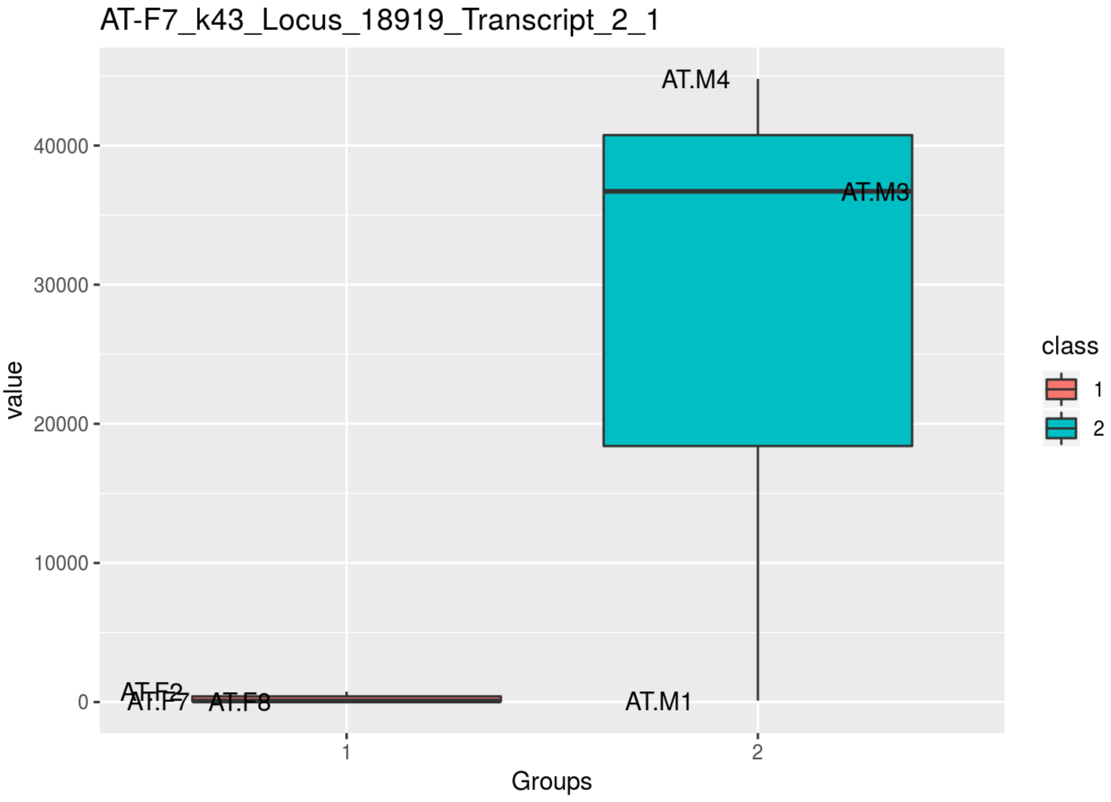

Supplementary Figure S5. Two-dimensional electrophoresis of male 6 (October) extract of urinary proteins. Spots 10-19 were cut out and analysed by nano-LC-MS/MS.

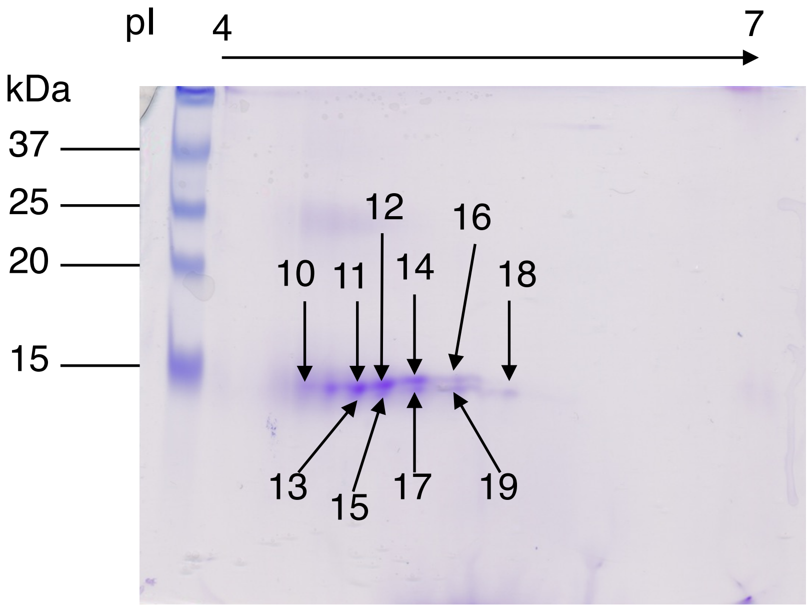

Supplementary Figure S6. MS/MS spectra of identified phosphopeptides from arvicolin. *m/z*, ion score, retention time and spectra of the naked peptides (A and C) and the corresponding phosphopeptides (B and D).

A: naked peptide (TSNMIYVYGKGQPLTPEQYEK)

Previous AA	Sequence	Next AA	PTM (AA position)	Score	Coverage		MH+	m/z	ppm	charge	Retention time
R	TSNMIYVYGKGQPLTPEQYEK	I		55.48	119	139	2445.19	816.07	3.80	3	14.1667

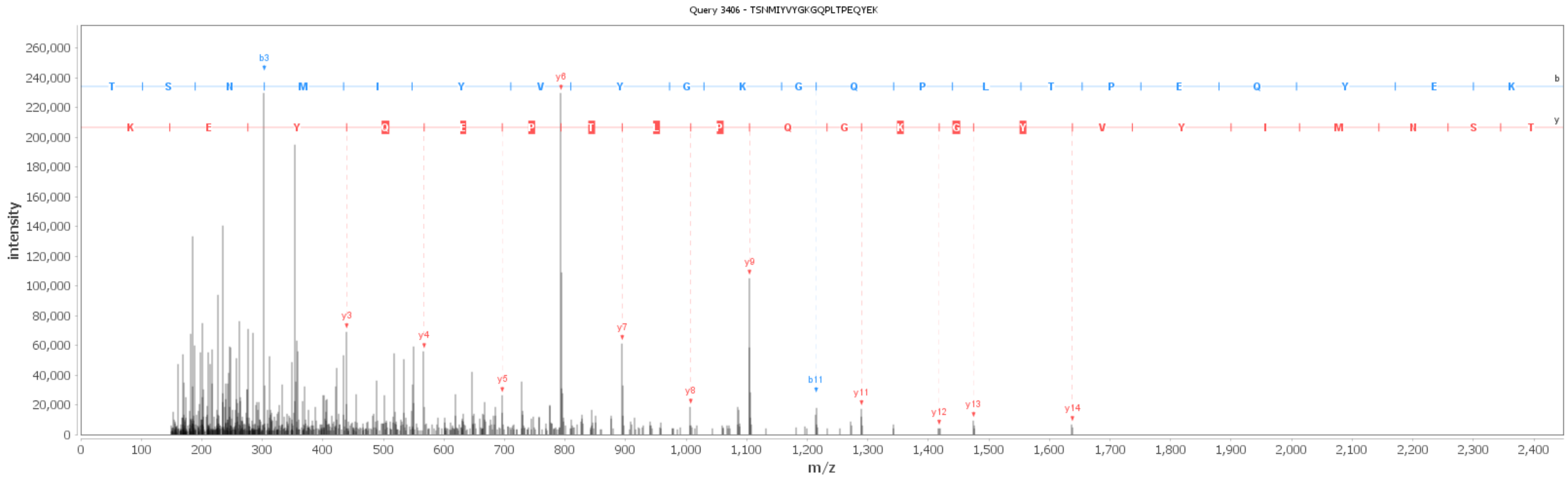

B: phosphorylated peptide (TSNMIYVYGKGQPLTPEQYEK)

Previous AA	Sequence	Next AA	PTM (AA position)	Score	Coverage		MH+	m/z	ppm	charge	Retention time
R	TSNMIYVYGKGQPLTPEQYEK	I	Phospho (S2); Phospho (Y8)	21.33	119	139	2605.12	869.43	62.02	3	12.5167
R	TSNMIYVYGKGQPLTPEQYEK	I	Phospho (T1); Phospho (Y6)	21.33	119	139	2605.12	869.43	62.02	3	12.5167
R	TSNMIYVYGKGQPLTPEQYEK	I	Phospho (T1); Phospho (Y8)	21.33	119	139	2605.12	869.43	62.02	3	12.5167
R	TSNMIYVYGKGQPLTPEQYEK	I	Phospho (S2); Phospho (Y6)	21.33	119	139	2605.12	869.43	62.02	3	12.5167

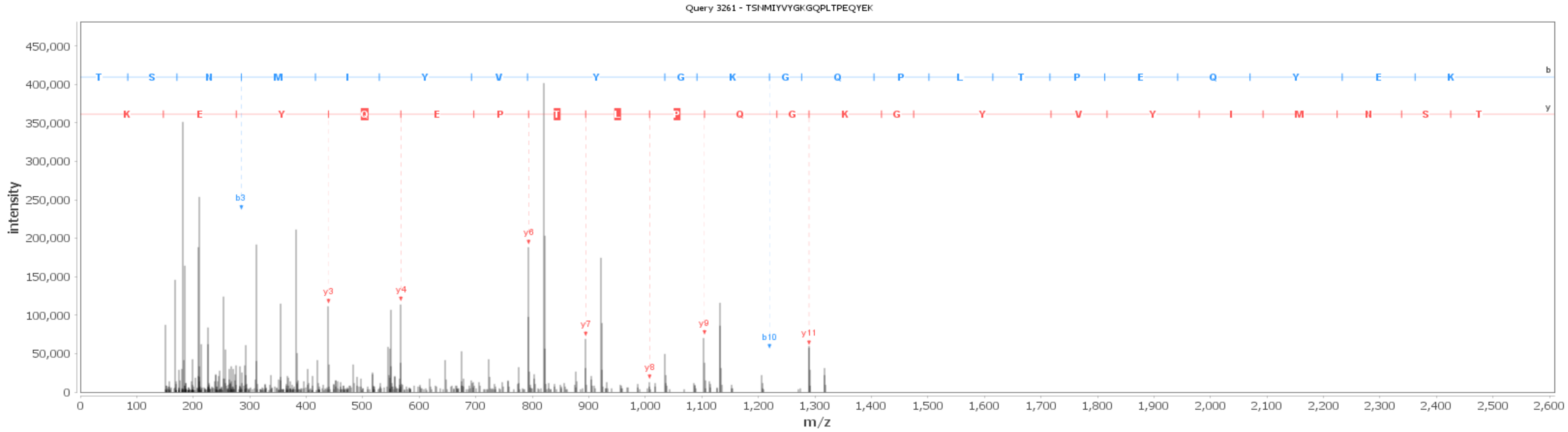

C: naked peptide (AQNIPTENIQDVLATDICPK)

Previous AA	Sequence	Next AA	PTM (AA position)	Score	Coverage		MH+	m/z	ppm	charge	Retention time
K	AQNIPTENIQDVLATDICPK	-	Carbamidomethyl (C18)	110.87	146	165	2239.12	1120.56	-2.55	2	17.9833

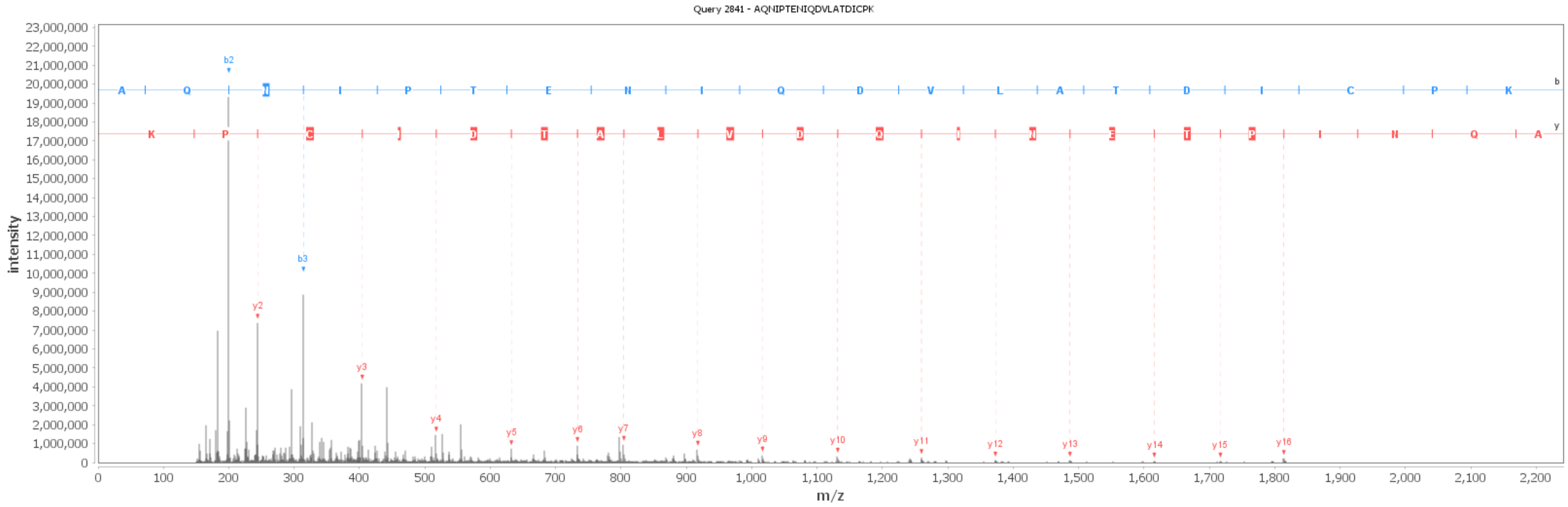

D: phosphorylated peptide (AQNIPTENIQDVLATDICPK)

Previous AA	Sequence	Next AA	PTM (AA position)	Score	Coverage		MH+	m/z	ppm	charge	Retention time
K	AQNIPTENIQDVLATDICPK	-	Phospho (T6); Carbamidomethyl (C18)	15.85	146	165	2319.08	1160.57	22.88	2	16.0167

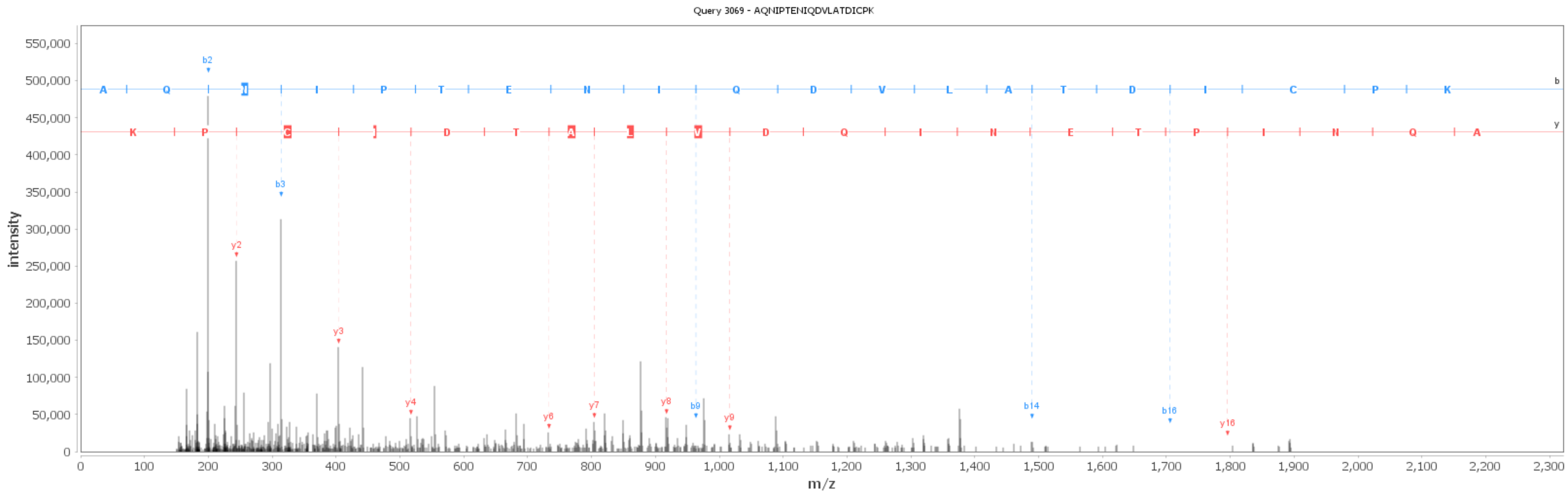

Supplement: Supplementary file 1 — Supplementary Information1 [file 41598_2019_54935_MOESM1_ESM.pdf]
