# Supplementary material for: Identification of potential chemosignals in the European water vole Arvicola terrestris
Source: Sci Rep. 2019 Dec 5;9:18378. doi: 10.1038/s41598-019-54935-z (PMC6895148; doi:10.1038/s41598-019-54935-z)
Supplement: Supplementary file 2 — Supplementary Information2 [file 41598_2019_54935_MOESM2_ESM.pdf]

Identification of potential chemical signals in the European water vole *Arvicola terrestris*

Patricia Nagnan-Le Meillour, Amandine Descamps, Chrystelle Le Danvic, Maurane Grandmougin, Jean-Michel Saliou, Christophe Klopp, Marine Milhes, Coralie Bompard, Didier Chesneau, Kevin Poissenot, Matthieu Keller

Supplementary information 2

The animals trapping is authorised in French departments of Cantal and Puy-de-Dôme by prefectoral orders 2015-1381 and 2015-155.

Liberté • Égalité • Fraternité
RÉPUBLIQUE FRANÇAISE

PREFET DE LA REGION AUVERGNE
PREFET DU PUY-DE-DOME

Arrêté N° 2015-155
portant sur l'obligation de lutte contre le campagnol terrestre (*Arvicola terrestris*) sur certaines communes du département du Puy-de-Dôme

Le Préfet de la Région Auvergne
Préfet du Puy de Dôme
Officier de la Légion d'honneur,
Chevalier de l'Ordre National du Mérite

Vu le règlement (CE) n° 1107/2009 du Parlement européen et du Conseil du 21 octobre 2009 concernant la mise sur le marché des produits phytopharmaceutiques et abrogeant les directives 79/117/CEE et 91/414/CEE du Conseil, notamment son article 67 ;

Vu le règlement d'exécution (UE) n° 540/2011 de la Commission du 25 mai 2011 portant application du règlement (CE) n° 1107/2009 du Parlement européen et du Conseil en ce qui concerne les substances actives approuvées, notamment la bromadiolone ;

Vu le code rural et de la pêche maritime, notamment les articles L.201-13, R.201-39 à R.201-43, et D.201-44 ;

Vu le code rural et de la pêche maritime, notamment les articles L.251-8 et L.253-7 ;

Vu le décret du 25 juillet 2013 portant nomination de M. Michel FUZEAU, Préfet de la région Auvergne, Préfet du Puy de Dôme ;

Vu le décret n° 2012-842 du 30 juin 2012 relatif à la reconnaissance des organismes à vocation sanitaire, des organisations vétérinaires à vocation technique, des associations sanitaires régionales ainsi qu'aux conditions de délégations de missions liées aux contrôles sanitaires ;

Vu l'arrêté ministériel du 13 mars 2014 portant reconnaissance des organismes à vocation sanitaire (OVS) dans le domaine animal et végétal, notamment la désignation de la FREDON Auvergne comme OVS pour le domaine végétal en région Auvergne ;

Vu l'arrêté du 31 juillet 2000 modifié établissant la liste des organismes nuisibles aux végétaux, produits végétaux et autres objets soumis à des mesures de lutte obligatoire ;

Vu l'arrêté du 14 mai 2014 relatif au contrôle des populations de campagnols nuisibles aux cultures ainsi qu'aux conditions d'emploi des produits phytopharmaceutiques contenant de la bromadiolone , et plus particulièrement son article 5 ;

Vu l'arrêté préfectoral n° 2015-153 du 23 octobre 2015 portant obligation de lutte contre le campagnol terrestre (*Arvicola terrestris*) sur certaines communes du département du Puy-de-Dôme ;

Vu le plan d'action régional de lutte contre le campagnol terrestre en Auvergne, pour lequel le conseil régional d'orientation de la politique sanitaire animal et végétal a émis un avis favorable, en sa séance du 25 juin 2015 ;

Vu la surveillance et les modalités d'organisation établies par la FREDON-FDGDON Auvergne et les structures ayant mis en place localement la lutte, et les exploitants agricoles qui se sont engagés dans cette lutte au travers de contrat de lutte, au sens de l'article 4 de l'arrêté du 14 mai 2014

susvisé, sur certaines communes du département du Puy de Dôme ;

Considérant que le campagnol terrestre (*Arvicola terrestris*) est classé comme danger sanitaire de deuxième catégorie au sens du décret n°2012-845 du 30 juin 2012 susvisé ;

Considérant que les cycles de pullulation de campagnols terrestres occasionnent, outre des désordres sanitaires, des pertes économiques importantes dans les exploitations agricoles touchées ;

Considérant que l'efficacité d'une lutte visant à la maîtrise des populations de ce rongeur réside essentiellement dans son caractère collectif, raisonné et précoce, au sein d'un territoire organisé pour cela, ainsi que la combinaison des méthodes entre elles, en particulier les méthodes préventives, le piégeage et des mesures favorisant la prédation ;

Considérant que le plan d'action régional susvisé donne la possibilité à la FREDON-FDGDON et à d'autres structures de mettre en place des plans de lutte et de surveiller les populations de campagnols terrestres ;

Sur proposition de la direction régionale de l'alimentation, de l'agriculture et de la forêt Auvergne,

ARRETE

Article 1 :

En application de l'article 5 de l'arrêté du 14 mai 2014 précité, et sans préjudice de mesures de restriction en matière de lutte susceptibles d'être instituées sur certaines zones ou à certaines périodes, la lutte contre le campagnol terrestre (*Arvicola terrestris*) est rendue obligatoire sur les zones concernées par la mise en œuvre du plan d'action régional campagnol susvisé, au sein des territoires des communes listées à l'annexe 1 du présent arrêté.

L'annexe 1 sera mise à jour en tant que de besoin.

Article 2 :

A ce titre, une organisation locale de lutte collective devra être mise en œuvre. Dans ce cadre, les exploitants devront s'engager dans un plan concerté d'actions.

Dans le cadre de l'organisation qui sera mise en place, les exploitants agricoles ou détenteurs de fonds situés au sein des territoires des communes listées à l'annexe 1 s'engagent dans des contrats et appliquent l'ensemble des mesures ainsi contractualisées.

Ils respectent tout particulièrement les consignes transmises par la FREDON-FDGDON ou la structure ayant mis en place localement le suivi de la lutte, en matière de précocité de surveillance et d'intervention.

Article 3 :

A défaut d'organisation locale mise en place, les exploitants agricoles ou détenteurs de fonds situés au sein des territoires des communes listées à l'annexe 1, participent obligatoirement à la mise en œuvre d'une lutte précoce, collective et raisonnée contre le campagnol terrestre, comme décrite à l'article 2 de l'arrêté du 14 mai 2014 susvisé.

Conformément à l'annexe I de l'arrêté du 14 mai 2014, ils s'assurent de la surveillance de leurs parcelles en lien avec la FREDON-FDGDON. Ils appliquent au moins deux méthodes de lutte alternative parmi celles listées à l'annexe I de l'arrêté du 14 mai 2014 et rappelées à l'annexe 2 du présent arrêté.

Article 4 :

Un comité départemental d'évaluation de la maîtrise des populations de campagnol terrestre est créé.

Ce comité est composé de la FREDON-FDGDON, de la chambre départementale d'agriculture, de la direction départementale des territoires, le cas échéant de la direction départementale en charge de la protection des populations pour l'impact sanitaire des campagnols, et pourra associer selon les sujets à aborder d'autres structures en qualité d'expert ou de sachant. Il sera présidé par le Préfet ou son représentant.

Ce comité a pour mission de suivre et d'évaluer la déclinaison du plan d'action régional dans le département, notamment de s'assurer que tout est en œuvre pour garantir la cohérence territoriale. En effet, la coordination des actions collectives sur un territoire donné est une garantie incontestable de réussite.

En ce sens, il vise à favoriser la mise en place de logiques de territoires selon les principes et les méthodes de l'arrêté du 14 mai 2014 en prenant en compte les problématiques qui peuvent être portées par les acteurs des territoires dans lesquels une organisation collective a été mise en place pour systématiser et concentrer les actions et ainsi accroître leur efficacité.

Ce comité pourra formuler toutes propositions utiles pour faire évoluer le plan d'action régional et sa déclinaison départementale.

Ce suivi départemental sera présenté en CROPSAV.

Article 5 :

La structure ayant mis en place la lutte informe la FREDON-FDGDON (qui est l'organisme à vocation sanitaire dans le domaine du végétal pour l'Auvergne) de l'organisation de la lutte et du déroulement des actions de lutte.

Article 6 :

Lorsque les exploitants ou détenteurs de fonds au sein des territoires des communes listées à l'annexe I utilisent des appâts traités à la bromadiolone, ils doivent se conformer à l'arrêté du 14 mai 2014 susvisé, chapitres II, III, IV et V, qui portent sur l'utilisation de ce produit phytopharmaceutique, sa traçabilité et l'information du public.

Article 7 :

La période d'obligation couverte par le présent arrêté s'étend de la date de sa signature au 31 décembre 2016.

Article 8 :

L'arrêté préfectoral du 23 octobre 2015 susvisé est abrogé et remplacé par le présent arrêté.

Article 9 :

Le présent arrêté peut être contesté sous deux mois à compter de sa publication, auprès du tribunal administratif compétent.

Article 10 :

Le secrétaire général de la préfecture du Puy-de-Dôme, le directeur départemental des territoires du Puy-de-Dôme et le directeur régional de l'alimentation de l'agriculture et de la forêt de Auvergne sont chargés, chacun en ce qui le concerne, de l'exécution du présent arrêté qui sera publié au recueil des actes administratifs de la préfecture du Puy de Dôme.

Fait à Clermont-Ferrand, le 28 OCT. 2015
Le Préfet,
P/Le Préfet et par délégation,
Le Secrétaire Général,

Thierry SUQUET

Annexe 1

Liste des communes où la lutte contre le campagnol terrestre (Arvicola terrestris) est rendue obligatoire :

Puy-de-Dôme :

- Canton d'Ambert

- Canton de Beaumont : commune de Saint-Genès-Champanelle

- Canton de Billom : communes de Bongheat, Egliseneuve-près-Billom, Estandeuil, Fayet-le-Chateau, Isserteaux, Montmorin, Neuville, Saint-Dier-d'Auvergne, Saint-Jean-des-Ollières, Saint-Julien-de-Coppel, Trézioux

- Canton de Brassac-les-Mines : communes de Anzat-le-Luguet, Apchat, Ardes, Augnat, Beaulieu, Champagnat-le-Jeune, La Chapelle-Marcousse, Chassagne, Dauzat-sur-Vodable, Egliseneuve-des-Liards, Esteil, La Godivelle, Madriat, Mazoires, Parentignat, Peslières, Les Pradeaux, Rentières, Roche-Charles-La-Mayrand, Saint-Alyre-es-Montagne, Saint-Etienne-sur-Usson, Saint-Genès-la-Tourette, Saint-Gervazy, Saint-Herent, Saint-Jean-en-Val, Saint-Jean-Saint-Gervais, Saint-Martin-d'Ollières, Saint-Quentin-sur-Sauxillanges, Sauxillanges, Sugères, Ternant-les-Eaux, Usson, Valz-sous-Chateauneuf, Vernet-la-Varenne

- Canton de Cébazat : commune de Sayat

- Canton de Chamalières : commune de Royat

- Canton de Châtel-Guyon : commune de Volvic

- Canton de Maringues : commune de Charnat

- Canton des Monts du Livradois

- Canton d'Orcines

- Canton de Saint-Eloy-les-Mines : communes d'Ars-les-Favets, Biollet, Charensat, Chateau-sur-Cher, Moureuille, Saint-Gervais-d'Auvergne, Saint-Hilaire, Saint-Priest-des-Champs, Sainte-Christine, Sauret-Besserve, Teilhet, Vergheas

- Canton de Saint-Georges-de-Mons : communes de Les Ancizes-Comps, Blot-l'Eglise, Champs, Charbonnières-les-Vieilles, Jozerand, Lisseuil, Loubeyrat, Manzat, Marcillat, Montcel, Pouzol, Saint-Angel, Saint-Gal-sur-Sioule, Saint-Georges-de-Mons, Saint-Hilaire-la-Croix, Saint-Pardoux, Saint-Quintin-sur-Sioule, Saint-Rémy-de-Blot, Vitrac

- Canton de Saint-Ours

- Canton du Sancy

- Canton de Thiers : communes de Celles-sur-Durolle, Palladuc, Sainte-Agathe, Saint-Victor-Montvianeix

- Canton de Vic-le-Comte : communes de Manglieu, Sallèdes

Annexe 2

Liste des méthodes de lutte alternative contre le campagnol terrestre (Arvicola terrestris)

Méthode de lutte	Objectif	Modalités
Lutte directe contre le campagnol	Diminuer les populations de campagnol	Piégeage
Lutte contre les taupes	Diminuer les habitats favorables au campagnol (galeries de taupes)	Piégeage (l'utilisation du phosphure d'hydrogène est possible mais réservé à un usage professionnel et spécifiquement agréé. L'utilisation de ce produit sort du cadre du présent arrêté)
Pratiques agricoles de travail du sol	Diminuer les habitats favorables au campagnol par destruction des galeries	Travail du sol par passage d'outils superficiels ou profonds dans le respect du cadre réglementaire de la PAC
Pratiques agricoles de pâture et fauche	Diminuer les habitats favorables au campagnol par destruction des galeries	Alternance fauche / pâture dans les prairies permanentes, accentuant la fréquence de piétinement du bétail, ou tout système le reproduisant
Pratiques agricoles de gestion de la couverture en herbe	Diminuer les habitats favorables au campagnol en réduisant les abris, les sources de nourriture et en favorisant la prédation naturelle	Broyage des refus, conduite des prairies en « gazon court », hersage, émoussage
Mesures d'entretien ou d'aménagement du paysage	Favoriser la prédation naturelle	Entretien ou plantation de haies, de murgers, de zones refuges pour les prédateurs naturels du campagnol
Mesures complémentaires à l'aménagement du paysage	Favoriser la prédation naturelle	Pose de nichoirs et/ou de perchoirs pour les prédateurs naturels du campagnol

Liberté • Égalité • Fraternité
RÉPUBLIQUE FRANÇAISE

PRÉFET DU CANTAL

RECUEIL DES ACTES

ADMINISTRATIFS

N° 57 du 26 octobre 2015

Cours Monthyon – B.P. 529 – 15005 AURILLAC CEDEX
Tel : 04 71 46 23 00 - Télécopie : 04 71 64 88 01 – Internet : http://www.cantal.gouv.fr

PREFET DU CANTAL

Arrêté N° 2015-1381 du 23 octobre 2015
portant sur l'obligation de lutte contre le campagnol terrestre (*Arvicola terrestris*) dans les communes du département du Cantal

Le Préfet du Cantal,
Chevalier de la Légion d'honneur,
Officier de l'Ordre National du Mérite,

Vu le règlement (CE) n° 1107/2009 du Parlement européen et du Conseil du 21 octobre 2009 concernant la mise sur le marché des produits phytopharmaceutiques et abrogeant les directives 79/117/CEE et 91/414/CEE du Conseil, notamment son article 67 ;

Vu le règlement d'exécution (UE) n° 540/2011 de la Commission du 25 mai 2011 portant application du règlement (CE) n° 1107/2009 du Parlement européen et du Conseil en ce qui concerne les substances actives approuvées, notamment la bromadiolone ;

Vu le code rural et de la pêche maritime, notamment les articles L.201-13, R. 201-39 à R. 201-43 et D.201-44 et les articles L. 251-8 et L. 253-7 ;

Vu le décret n° 2012-842 du 30 juin 2012 relatif à la reconnaissance des organismes à vocation sanitaire, des organisations vétérinaires à vocation technique, des associations sanitaires régionales ainsi qu'aux conditions de délégations de missions liées aux contrôles sanitaires ;

Vu le décret du 18 septembre 2014 portant nomination du préfet du Cantal, Monsieur Richard VIGNON ;

Vu l'arrêté du 31 juillet 2000 modifié établissant la liste des organismes nuisibles aux végétaux, produits végétaux et autres objets soumis à des mesures de lutte obligatoire ;

Vu l'arrêté ministériel du 13 mars 2014 portant reconnaissance des organismes à vocation sanitaire (OVS) dans le domaine animal et végétal, notamment la désignation de la FREDON Auvergne comme OVS pour le domaine végétal en région Auvergne ;

Vu l'arrêté du 14 mai 2014 relatif au contrôle des populations de campagnols nuisibles aux cultures ainsi qu'aux conditions d'emploi des produits phytopharmaceutiques contenant de la bromadiolone, et plus particulièrement son article 5 ;

Vu le Plan d'Actions Régional de lutte contre le campagnol en Auvergne (« PAR Campagnol » en Auvergne) et l'avis favorable des membres du CROPSAV (conseil régional d'orientation de la politique sanitaire animale et végétale), section végétale, de l'Auvergne sur la mise en œuvre de ce plan, en séance plénière du 25 juin 2015 ;

Considérant que le campagnol terrestre (*Arvicola terrestris*) est réputé classé comme danger sanitaire de deuxième catégorie au sens du décret n°2012-845 du 30 juin 2012 ;

Considérant que les cycles de pullulation de campagnols terrestres occasionnent, outre des dangers sanitaires, des pertes économiques importantes dans les exploitations agricoles touchées ;

Considérant que l'efficacité d'une lutte visant à la maîtrise des populations de rongeurs réside essentiellement dans son caractère collectif, raisonné et précoce, au sein d'un territoire organisé pour cela , ainsi que la combinaison des méthodes entre elles, en particulier les méthodes préventives, le piégeage et des mesures favorisant la prédation ;

Considérant l'avis du CROPSAV sur le plan d'actions régional qui donne la possibilité à la FREDON, aux FDGDON et à d'autres structures de mettre en place des plans de lutte et de surveiller les populations de campagnols terrestres ;

Sur proposition du Directeur régional de l'alimentation, de l'agriculture et de la forêt d' Auvergne, à partir des demandes reçues,

ARRETE

Article 1 :

En application de l'article 5 de l'arrêté du 14 mai 2014 précité, et sans préjudice de mesures de restriction en matière de lutte susceptibles d'être instituées sur certaines zones ou à certaines périodes, la lutte contre le campagnol terrestre (*Arvicola terrestris*) est rendue obligatoire dans toutes les communes du Cantal.

Article 2 :

À ce titre une organisation locale de lutte collective devra être mise en œuvre. Dans ce cadre, les exploitants devront s'engager dans un plan concerté d'actions.

Dans le cadre de l'organisation qui sera mise en place, les exploitants agricoles ou détenteurs de fonds s'engagent dans des contrats et appliquent l'ensemble des mesures ainsi contractualisées.

Ils respectent tout particulièrement les consignes transmises par la FREDON et la FDGDON du Cantal ou la structure ayant mis en place localement le suivi de la lutte, en matière de précocité de surveillance et d'intervention.

Article 3 :

À défaut d'organisation locale mise en place, les exploitants agricoles ou détenteurs de fonds participent obligatoirement à la mise en œuvre d'une lutte précoce, collective et raisonnée contre le campagnol terrestre, comme décrite à l'article 2 de l'arrêté du 14 mai 2014.

Conformément à l'annexe I de l'arrêté du 14 mai 2014, ils s'assurent de la surveillance de leurs parcelles, en lien avec la FREDON et la FDGDON du Cantal. Ils appliquent au moins deux méthodes de lutte alternative parmi celles listées à l'annexe I de l'arrêté du 14 mai 2014 et rappelées à l'annexe du présent arrêté.

Article 4 :

Un comité départemental d'évaluation de la maîtrise des populations de campagnol terrestre est créé.

Ce comité sera composé de la FREDON ou de la FDGDON, de la Chambre départementale d'agriculture, de la Direction Départementale des Territoires, de la Direction Départementale de la Cohésion sociale et de la Protection des Populations pour l'impact sanitaire des campagnols. Il pourra associer selon les sujets à aborder d'autres structures en qualité d'expert ou de sachant. Il sera présidé par le Préfet ou son représentant.

Ce comité a pour mission de suivre et d'évaluer la déclinaison du plan d'actions régional dans le département, notamment de s'assurer que tout est en œuvre pour garantir la cohérence territoriale. En effet, la coordination des actions collectives sur un territoire donné est une garantie incontestable de réussite.

En ce sens, il vise à favoriser la mise en place de logiques de territoires selon les principes et les méthodes de l'arrêté du 14 mai 2014 en prenant en compte les problématiques qui peuvent être portées par les acteurs des territoires dans lesquels une organisation collective a été mise en place pour systématiser et concentrer les actions et ainsi accroître leur efficacité.

Ce comité pourra formuler toutes propositions utiles pour faire évoluer le plan d'actions régional et sa déclinaison départementale.

Ce suivi départemental sera présenté en CROPSAV.

Article 5 :

La structure ayant mis en place la lutte informe la FREDON d'Auvergne qui est l'organisme à vocation sanitaire dans le domaine du végétal pour l'Auvergne, lors de l'organisation de la lutte et au cours du déroulement des actions de lutte.

Article 6 :

Lorsque les exploitants ou détenteurs de fonds utilisent des appâts traités à la bromadiolone, ils doivent se conformer à l'arrêté du 14 mai 2014, chapitre II, III, IV et V, qui portent sur l'utilisation de ces produits phytosanitaires, leur traçabilité et l'information du public.

Article 7 :

La période d'obligation couverte par le présent arrêté s'étend de la date de sa signature au 31 décembre 2016.

Article 8 :

Le présent arrêté peut faire l'objet d'un recours gracieux auprès du préfet du Cantal, d'un recours hiérarchique auprès du ministère concerné, d'un recours contentieux auprès du tribunal administratif de Clermont-Ferrand, dans un délai de deux mois à compter de la publication du présent arrêté.

Article 9 :

Le secrétaire général de la préfecture du Cantal, le directeur départemental des territoires et la directrice départementale de la cohésion sociale et de la protection des populations sont chargés, chacun en ce qui le concerne, de l'exécution du présent arrêté qui sera publié au recueil des actes administratifs de la préfecture du département du Cantal.

Fait à Aurillac le 23 octobre 2015

Le Préfet du Cantal,

signé

Richard VIGNON

Annexe à l’arrêté préfectoral N°2015-1381 du 23 octobre 2015

Liste des méthodes de lutte alternative contre le campagnol terrestre (*Arvicola terrestris*)

Méthode de lutte	Objectif	Modalités
Lutte directe contre le campagnol	Diminuer les populations de campagnol	Piégeage
Lutte contre les taupes	Diminuer les habitats favorables au campagnol (galeries de taupes)	Piégeage (l'utilisation du phosphure d'hydrogène est possible mais réservé à un usage professionnel et spécifiquement agréé. L'utilisation de ce produit sort du cadre du présent arrêté)
Pratiques agricoles de travail du sol	Diminuer les habitats favorables au campagnol par destruction des galeries	Travail du sol par passage d'outils superficiels ou profonds dans le respect du cadre réglementaire de la PAC
Pratiques agricoles de pâture et fauche	Diminuer les habitats favorables au campagnol par destruction des galeries	Alternance fauche / pâture dans les prairies permanentes, accentuant la fréquence de piétinement du bétail, ou tout système le reproduisant
Pratiques agricoles de gestion de la couverture en herbe	Diminuer les habitats favorables au campagnol en réduisant les abris, les sources de nourriture et en favorisant la prédation naturelle	Broyage des refus, conduite des prairies en « gazon court », hersage, émoussage
Mesures d'entretien ou d'aménagement du paysage	Favoriser la prédation naturelle	Entretien ou plantation de haies, de murgers, de zones refuges pour les prédateurs naturels du campagnol
Mesures complémentaires à l'aménagement du paysage	Favoriser la prédation naturelle	Pose de nichoirs et/ou de perchoirs pour les prédateurs naturels du campagnol
